# Hindgut microbiota in laboratory-reared and wild *Triatoma infestans*

**DOI:** 10.1371/journal.pntd.0007383

**Published:** 2019-05-06

**Authors:** Andreea Waltmann, Alexandra C. Willcox, Sujata Balasubramanian, Katty Borrini Mayori, Sandra Mendoza Guerrero, Renzo S. Salazar Sanchez, Jeffrey Roach, Carlos Condori Pino, Robert H. Gilman, Caryn Bern, Jonathan J. Juliano, Michael Z. Levy, Steven R. Meshnick, Natalie M. Bowman

**Affiliations:** 1 Institute for Global Health and Infectious Diseases, School of Medicine, University of North Carolina at Chapel Hill, Chapel Hill, North Carolina, United States of America; 2 Department of Epidemiology, Gillings School of Global Public Health, University of North Carolina at Chapel Hill, Chapel Hill, North Carolina, United States of America; 3 Zoonotic Disease Research Laboratory, Unidad de Una Salud, Universidad Peruana Cayetano Heredia, Arequipa, Perú; 4 Microbiome Core Facility, School of Medicine, University of North Carolina at Chapel Hill, Chapel Hill, North Carolina, United States of America; 5 Department of International Health, Johns Hopkins Bloomberg School of Public Health, Baltimore, Maryland, United States of America; 6 Department of Epidemiology and Biostatistics, School of Medicine, University of California-San Francisco, San Francisco, California, United States of America; 7 Division of Infectious Diseases, School of Medicine, University of North Carolina at Chapel Hill, Chapel Hill, North Carolina, United States of America; 8 Department of Biostatistics, Epidemiology & Informatics, University of Pennsylvania Perelman School of Medicine, Philadelphia, Pennsylvania, United States of America; Universidad de Buenos Aires, ARGENTINA

## Abstract

Triatomine vectors transmit *Trypanosoma cruzi*, the etiological agent of Chagas disease in humans. Transmission to humans typically occurs when contaminated triatomine feces come in contact with the bite site or mucosal membranes. In the Southern Cone of South America, where the highest burden of disease exists, *Triatoma infestans* is the principal vector for *T*. *cruzi*. Recent studies of other vector-borne illnesses have shown that arthropod microbiota influences the ability of infectious agents to colonize the insect vector and transmit to the human host. This has garnered attention as a potential control strategy against *T*. *cruzi*, as vector control is the main tool of Chagas disease prevention. Here we characterized the microbiota in *T*. *infestans* feces of both wild-caught and laboratory-reared insects and examined the relationship between microbial composition and *T*. *cruzi* infection using highly sensitive high-throughput sequencing technology to sequence the V3-V4 region of the 16S ribosomal RNA gene on the MiSeq Illumina platform. We collected 59 wild (9 with *T*. *cruzi* infection) and 10 lab-reared *T*. *infestans* (4 with *T*. *cruzi* infection) from the endemic area of Arequipa, Perú. Wild *T*. *infestans* had greater hindgut bacterial diversity than laboratory-reared bugs. Microbiota of lab insects comprised a subset of those identified in their wild counterparts, with 96 of the total 124 genera also observed in laboratory-reared insects. Among wild insects, variation in bacterial composition was observed, but time and location of collection and development stage did not explain this variation. *T*. *cruzi* infection in lab insects did not affect α- or β-diversity; however, we did find that the β-diversity of wild insects differed if they were infected with *T*. *cruzi* and identified 10 specific taxa that had significantly different relative abundances in infected *vs*. uninfected wild *T*. *infestans* (*Bosea*, *Mesorhizobium*, *Dietzia*, and *Cupriavidus* were underrepresented in infected bugs; *Sporosarcina*, an unclassified genus of *Porphyromonadaceae*, *Nestenrenkonia*, *Alkalibacterium*, *Peptoniphilus*, *Marinilactibacillus* were overrepresented in infected bugs). Our findings suggest that *T*. *cruzi* infection is associated with the microbiota of *T*. *infestans* and that inferring the microbiota of wild *T*. *infestans* may not be possible through sampling of *T*. *infestans* reared in the insectary.

## Author summary

Chagas disease in humans is caused by the parasite *Trypanosoma cruzi* and it is endemic to the Americas. Poor populations are most at risk. The parasite infects an estimated six million people of 21 endemic countries in the Americas, with 30,000 new infections yearly. The main mode of transmission is vector-borne by triatomine bugs, which tend to live in close association with humans. The main Chagas disease vector in the Southern Cone of South America, where the highest burden of disease exists, is *Triatoma infestans*. As blood-sucking insects, triatomines become infected when they bite a *T*. *cruzi*-positive human and once infected they transmit the parasites in their feces. Controlling the vector populations is the main strategy of Chagas disease transmission reduction efforts. Microbiota-mediated methods to control this vector-borne disease are now being explored to determine whether microbes typically found in the vectors’ gut have a detrimental effect on *T*. *cruzi* and how they may be used to modify the vector and curb the ability for *T*. *cruzi* to be transmitted to humans. To advance this new field, we first must gain better knowledge of the gut microbiota of triatomines. Our study is the first to use sensitive high-throughput methods to study the gut microbes of *T*. *infestans*, using both laboratory-reared and wild insects. We have found that the microbial composition of *T*. *infestans* in the laboratory does not reflect the complete collection of gut microbes of wild *T*. *infestans* and inferring the gut microbiota profile of wild insects through studying lab insects alone may not be possible. We also found evidence that in wild insects *T*. *cruzi* affects the composition of the gut microbiota and identified some bacterial taxa which may be important in modulating the *T*.*infestans*-*T*.*cruzi* relationship.

## Introduction

Chagas disease, caused by the protozoan parasite *Trypanosoma cruzi*, infects an estimated six million people residing in 21 endemic countries in the Americas, with 30,000 new infections yearly [1]. The most important method of *T*. *cruzi* transmission to humans is vector-borne: *T*. *cruzi* undergoes development exclusively in the gut of hematophagous triatomine species, and when the vector defecates during its blood meal, the parasite is inoculated via the bite site or mucosal surfaces [2]. Of the seven main vectors of *T*. *cruzi* across the Americas [3, 4], *Triatoma infestans* (Klug, 1834) is the most important vector of Chagas disease in the Southern Cone of South America, where the insect lives in close association with human dwellings and domestic animals.

Growing evidence suggests that the gut microbiota of insect vectors can affect the ability of human pathogens to colonize and persist in the vector or alter the vectors’ competence to transmit pathogens to the human host [5–8] by modulating insect immune responses or competing for resources or producing inhibitory molecules [6, 9–12]. Therefore, to take advantage of these processes, manipulation of insect microbiota is becoming a novel avenue for controlling the spread of vector-borne infections [13–15]. Coprophagy (i.e. the ingestion of feces within the insects’ colonies) is a principal process by which symbiont intestinal microbes are acquired [16, 17]. To date, all symbionts of triatomines belong to the *Actinomycetes* group [18]. In the wild, acquisition of non-symbiotic/environmental or host-associated microbes can also occur through coprophagy and during bloodmeals when in contact with the mammalian host [19]. The intestinal microbial composition has been shown to be affected by taking a blood meal [17] and blood meal source [20]. Because *T*. *cruzi* develops in the gut of triatomines (and more specifically for *T*. *infestans* this development takes place in the rectum [21]) and is expected to interact with gut microbes, several approaches to control *T*. *cruzi* infection via alterations of the triatomine gut microbiota have been explored [16, 22]. For example, Beard *et al* leveraged the coprophagic habits of *Rhodnius prolixus*, another important triatomine vector of *T*. *cruzi*, to replace actinobacterial *Rhodococcus rhodnii* symbionts found ubiquitously in adult bugs with paratransgenic *R*. *rhodnii* carrying trypanocidal genes [16]. Another group utilized genetically modified *R*. *rhodnii* and *Escherichia coli* to induce RNA interference to affect redox state in the gut and reduce vector fitness [22]. Development of these and other novel vector control strategies is dependent upon a better understanding of the gut microbiome of triatomines and their relationship to *T*. *cruzi* infection and vector competence.

Studies of triatomine microbiota to date have been limited by small sample size and insensitive technology, such as culture-dependent methods, denaturing gradient gel electrophoresis or conventional PCR [17, 23, 24], though more recent high-throughput deep sequencing studies have been published [20, 25–27]. In addition, many of these studies have predominantly focused on laboratory-reared insects only. The microbiome of triatomines have typically been found to be of low complexity, with only a few dominant genera which are not always the same across species, feeding status, blood meal source, or intestinal region sampled. Microbiota of wild insects has also been found to differ from laboratory insects [17, 20, 23, 24, 26–30]. Similarly, studies which investigated the interaction between *T*. *cruzi* and the triatomine gut microbiota have found variable effects, likely modulated by vector species, geographic location, development stage and sex, blood meal source, and other unknown confounders. In the lab, gut symbionts of *Rhodnius prolixus* have been shown to be disrupted by *T*. *cruzi*, as seen in the reduction of *Serratia marcescens* strains, which are associated with some triatomine species and have trypanolytic activity [31]. Triatomines, and more specifically *Rhodnius prolixus*, were the pioneering effort in demonstrating that manipulation of insect resident microbiota by the use of a paratransgenic vector expressing trypanocidal genes can thwart the development of a human pathogen [16]. Although microbiota-based efforts to control *T*. *cruzi* transmission are showing hints of promise, they are hampered by a solid knowledge of the triatomine microbiota.

To our knowledge, there have been no examinations of the microbiome of wild *Triatoma infestans* by high-throughput sequencing, and there is only one study examining eight lab-reared *T*. *infestans* [25]. Using deep sequencing of the 16S ribosomal RNA gene at the variable V3-V4 locus we characterized the microbiome of 59 wild-caught and 10 laboratory-reared *T*. *infestans* from endemic communities in Arequipa, Perú. The objective of our study was to identify and compare the hindgut microbial composition of lab-reared and wild *T*. *infestans* and of *T*. *cruzi*-infected and uninfected insects and explore correlations between *T*. *cruzi* infection and specific microbiota profiles.

## Methods

### Ethics statement

The Institutional Animal Care and Use Committee (IACUC) of the Universidad Peruana Cayetano Heredia reviewed and approved the entomological survey and house inspections protocol used for this study (identification number 61287) and the triatomine colonies maintenance protocol (identification number 62782). The committee is registered with the United States National Institutes of Health (PHS Approved Animal Welfare Assurance Number A5146-01) and adheres to the Animal Welfare Act of 1990 (identification number 61287). The committee is registered with the United States National Institutes of Health (PHS Approved Animal Welfare Assurance Number A5146-01) and adheres to the Animal Welfare Act of 1990.

### Insect rearing and capture

Insect rearing and mouse work were performed as described in Salazar *et*. *al* (2015) [32]. Laboratory triatomines (n = 20) were reared in closed cages from eggs sourced from laboratory insect colonies maintained since 2008 at the Zoonotic Disease Research Laboratory, Unidad de Una Salud, Universidad Peruana Cayetano Heredia in Arequipa, Perú. The original colonies were initiated with eggs of local *T*. *infestans* from Arequipa. The insectary was located in an urban neighborhood of Arequipa (altitude 2300 m above sea level). Chickens and mice used to feed the bugs were housed in an enclosed patio next to the insectary. Triatomines are not found in the neighborhood surrounding the laboratory. Approximately 21 days after hatching, the 20 insects were fed on chickens on which adult *T*. *infestans* had previously fed (the adults were removed prior to the feeding of young insects), resulting in indirect coprophagy. Later, third instar stage insects were fed on either *T*. *cruzi*-infected or *T*. *cruzi*-negative BALB/c mice: 10 of the 20 triatomines were fed to repletion on BALB/c and the other 10 fed to repletion on uninfected BALB/c mice. Prior to insect feeding, the mice received an intraperitoneal inoculation with 10^3^ *T*. *cruzi* parasites/100 μl of a local strain, confirmed as Discrete Typing Unit 1 using a published PCR-RFLP protocol [32] and were confirmed to be *T*. *cruzi* positive or negative by microscopy of whole blood. 45 days after this blood meal, with no interval blood-feeding, triatomines were examined for *T*. *cruzi* infection by microscopy and their fecal sample collected. Only the insects which met the following criteria were sampled: i) had fed to repletion to ensure the probability of *T*. *cruzi* infection and guarantee molting to the fourth instar stage; ii) had molted to fourth instar. Only insects that had *T*. *cruzi* status confirmed by fecal microscopy were included for further analyses. Three additional lab insects from the same colony were fed to repletion on uninfected mice in 2015 using the same rearing and feeding protocol to increase the sample size of our uninfected controls. In total for this study, we had 7 laboratory-reared insects from August 2013 (n uninfected = 3 bugs, n infected = 4) and 3 uninfected laboratory-reared insects from August 2015) (S1 Table).

Wild triatomines (n = 59) were captured by our research team and the Arequipa Ministry of Health vector control personnel as part of routine surveillance and treatment activities between November 2011 and May 2012 and in August 2015. Of 59 insects, 53 (89.8%) were collected in urban and periurban districts of the city of Arequipa (Alto Selva Alegre, Alegre, Cayma, Hunter, Tiabaya and Yura) and the remainder (n = 6, 10.2%) from the rural district of Murco, located approximately 100 kilometers from the city of Arequipa. Triatomines were collected from household or peridomestic areas, including animal pens, after application of deltamethrin to these surfaces. Live triatomines were transported to the laboratory and analyzed for *T*. *cruzi* parasites by fecal microscopy on the day of capture.

### Collection and examination of *T*. *infestans* fecal samples

Wild triatomines were examined on the day of capture while still alive at the Arequipa insectary. Laboratory-reared bugs were examined and fecal material collected 45 days after last blood meal. A drop of fecal material was expressed onto a glass slide by applying gentle pressure to the bug’s abdomen. Fecal material was mixed with a drop of saline solution and examined by light microscopy at 400X for parasites by an experienced microscopist. Taking care to avoid contact between the insect and the paper, 2–5 drops of triatomine fecal material were expressed from the same bug by gentle abdominal pressure using tweezers onto Whatman filter paper (grade 3MM, catalog number 3030–347), allowed to air-dry completely at ambient temperature on the benchtop, and stored frozen in sealed plastic bags containing sterile silica desiccant packets at -20°C until cold-chain shipment and further laboratory procedures at the University of North Carolina at Chapel Hill, United States.

### DNA extraction and *T*. *cruzi* PCR

DNA was extracted using a modified phenol-chloroform technique at the University of North Carolina at Chapel Hill. Initially, fecal spots were cut out of the filter paper with razor blades cleaned with 70% ethanol and placed in sterile snap-cap vials containing 180 μl of sterile Tail lysis buffer (50 mM Tris titrated to pH 7.5, 100 mM EDTA, 100 mM NaCl, 1% SDS) with 20 μl proteinase K. The specimens were incubated overnight at 50°C, then heated to 90°C for 5 minutes. After a brief centrifugation step to pellet the filter paper, the supernatant was diluted to 200 μl in sterile TE buffer and used for DNA extraction using a published phenol-chloroform extraction [33] in a fume hood with surfaces which were thoroughly cleaned with 70% ethanol and DNAZap (Thermo Fisher Scientific) decontaminant spray prior to specimen handling. DNA extraction negative controls were included (empty snap-cap tubes to which Tail lysis buffer and all subsequent DNA extraction reagents were added) and we used the High Sensitivity dsDNA Qubit assay (Thermo Fisher Scientific) to quantify the amount of contaminating DNA. The assay did not show a detectable level of double-stranded DNA and thus these negative controls were not included in the V3-V4 16S amplicon generation steps.

*T*. *cruzi* infection status for each bug was confirmed using quantitative real time polymerase chain reaction (qPCR) on an Applied Biosystems Viia 7 real-time PCR machine with the published primers Cruzi 1 (5'-ASTCGGCTGATCGTTTTCGA-3’) and Cruzi2 (5'-AATTCCTCCAAGCAGCGGATA-3’) and the probe Cruzi3 (FAM- CACACACTGGACACCAA-NFQ-MGB) based on a published protocol [34]. In brief, 0.5 μl of Cruzi3 probe at 25 μM concentration and 0.5 μl each of Cruzi1 and Cruzi2 primers at 90 μM concentration were mixed with 5 μl template DNA, 18.5 μl water, and 25 μl ROX FastStart Universal Master Mix (Roche) and amplified under the following conditions: 50°C for 2 minutes, 95°C for 10 minutes, then 40 cycles of 95°C for 15 seconds followed by 58°C for 60 seconds.

### Triatomine microbiome library preparation and sequencing

The V3-V4 region of the 16S rRNA gene was amplified using published primers [35], producing amplicons of approximately 460 base pairs. Amplicon generation steps were performed in a laminar flow hood with surfaces sprayed with 70% ethanol and DNAZap decontaminant. To further reduce the likelihood of contamination from the laboratory environment, we used UV-irradiated plasticware and reagents (with the exception of the Taq polymerase). PCR negative controls were included and contained all PCR components to which sterile water was added in place of specimen DNA. To facilitate multiplexing and pooling of amplicons during sequencing library preparation, a 10-nucleotide Multiplex Identifier (MID) barcode sequence was added to the 5′ end of the forward primer, a method previously validated by our group [36]. Fragments were amplified using reagents from the KAPA HiFi HotStart PCR Kit (Kapa Bioystems) under the following conditions: initial denaturation for 3 minutes at 95°C; 35 cycles of 98°C for 20 seconds, 55°C for 30 seconds, and 72°C for 30 seconds; and final extension at 72°C for 5 minutes. PCR products were visualized on a 1% agarose gel. PCR products were then purified using the Purelink Pro PCR cleanup kit (Thermo Fisher Scientific) and quantified using the QuantIT Picogreen dsDNA assay kit (Thermo Fisher Scientific), after which they were pooled in equimolar amounts. The quantification assay did not detect contaminating DNA in our PCR negative controls, and as a result these were not included in the amplicon pool for sequencing. Illumina barcoded index adapters (Bioo Scientific) were ligated onto the pooled fragments after end filling and A tailing using Klenow and ligase (Enzymatics). Resulting libraries were amplified for 8 cycles using the KAPA Library Amplification Kit (Kapa Biosystems), cleaned using Kapa Pure beads (Kapa Biosystems) at a ratio of 0.6:1 beads to DNA following manufacturer instructions. Libraries were quantified using the Agilent 4200 TapeStation (Agilent Technologies), pooled in equimolar amounts, then sequenced on the Illumina MiSeq platform by a paired-end 2x250 sequencing run at the High Throughput Sequencing Facility at the University of North Carolina at Chapel Hill.

### Bioinformatic processing

Raw Illumina FASTQ sequences were first inspected by the quality control software FastQC [37]. Next, the sequences were processed and analyzed in the QIIME software package (v 1.9.1) [38]. The forward and reverse paired-end FASTQ reads were joined by invocation of the fastq-join with default parameters. A custom demultiplex step was applied to accommodate our two-index data structure in each read (i.e. library-level index and sample-level 10 base pair barcode). Linker primer sequences and sample-level barcodes were trimmed. Low-quality sequences where 70% of the base pairs were below a Phred score of 24 were excluded. Operational Taxonomic Units (OTUs) were then defined with the uclust algorithm and the open reference approach [39] at a similarity threshold of 97%, followed by taxonomy assignment with respect to the GreenGenes database release 13.8 [40]. Sequences were aligned using PyNAST [41] and a phylogenetic tree was built with FastTree 2.1.3 [42]. The OTUs retained for downstream analyses passed the following quality filters: (i) a minimum OTU size of 10 sequences across all samples, (ii) a filtering depth of 0.01% (i.e. any OTU making up less than 0.01% of the total observation count was excluded), (iii) singleton OTUs (i.e. observed in less than 2 samples) were excluded. If after the GreenGenes taxonomic assignment a taxon of interest was ambiguous at the genus level we then consulted the 16S sequences in the National Center for Biotechnology Information (NCBI) Genbank repository with the Basic Local Alignment Search Tool (BLAST) [43].

### Microbial composition analyses

Diversity estimates (α-diversity, within-group, and β-diversity, between group) were calculated with respect to origin (wild-caught *vs*. laboratory-reared *T*. *infestans*), *T*. *cruzi* infection, stage, and temporal and spatial factors. We estimated α-diversity analyses, including non-phylogenetic (observed number of taxa and Chao1) and phylogenetic (Faith’s Phylogenetic Diversity) metrics were estimated using QIIME [38] at minimum sampling depths of 2000 sequences per subsample. We compared α-diversity between sample groups with non-parametric two-sample *t*-tests using 1000 Monte Carlo permutations to calculate the *p*-values.

β-diversity was estimated in QIIME [38] using abundance-based Bray-Curtis, occurrence-based unweighted Unifrac [44] and quantitative abundance and phylogeny-based weighted Unifrac [45] at a minimum sub-sampling depth of 2000 sequences. Results were summarized and visualized through principal coordinate analysis (PCoA) implemented in QIIME [38]. To test whether sample groups were statistically different we used non-parametric ANOSIM (ANalysis Of Similarities) tests [46] and non-parametric two-sample *t*-tests with 1000 Monte Carlo permutations to derive *p*-values. To test correlations between sample groups and distribution of taxa, we computed Spearman’s Rho in QIIME [38] with bootstrapping and 1000 permutations by invocating the observation_metadata_correlation.py pipeline. To analyze which taxa were differentially abundant between samples groups, we employed two different methods: 1) non-parametric Kruskal-Wallis tests comparing the relative abundance of each taxon between sample groups with 1000 Monte Carlo permutations and Bonferroni-corrections to derive *p*-values and 2) with the DESeq2 method implemented in the differential_abundance.py pipeline [47] (package version 1.18.1). DESeq2 makes use of negative binomial generalized linear models and incorporates data-driven prior distributions to estimate dispersion and logarithmic fold changes between sample groupings [47]. Graphical visualizations were done with Graphpad PRISM (La Jolla California, USA) and with *R* package ggplot2.

## Results

### Insect collection and *T*. *cruzi* qPCR

Fecal specimens from 69 *T*. *infestans* (59 wild-caught and 10 laboratory-reared) were used in this study. Characteristics of these 69 insects are presented in S1 Table. Nine of the 59 wild bugs (15.3%) and 4 of the 10 laboratory bugs (40.0%) were *T*. *cruzi-*infected, confirmed by both microscopy of fresh fecal samples and qRT-PCR. Approximately half of the wild insects (32, 54.2%) were collected in the urban district of Alto Selva Alegre, but the majority of infected wild insects were captured in the rural district of Murco (5/9, 55.6%). Of 59 wild insects, 29 were adults (49.2%), 17 (28.8%) were fifth instars, 7 (11.9%) were fourth instars and the remainder 6 insects (10.2%) were third instars. Six of the 9 infected wild triatomines were fifth instars and the other 3 were adults. No wild infected third and fourth instars were sampled. Further sample details can be found in S1 Table.

### Deep sequencing of 16S rRNA gene amplicons

The V3-V4 region of the 16S rRNA gene was amplified and sequenced from the extracted DNA of all bugs. A total 2,051,172 reads were obtained from Illumina MiSeq platform with paired-end reads of 250 base pairs. The joined reads have been deposited in the Sequence Read Archive (SRA) under the accession PRJNA527262.

### Taxonomic determination

**Fig 1:**
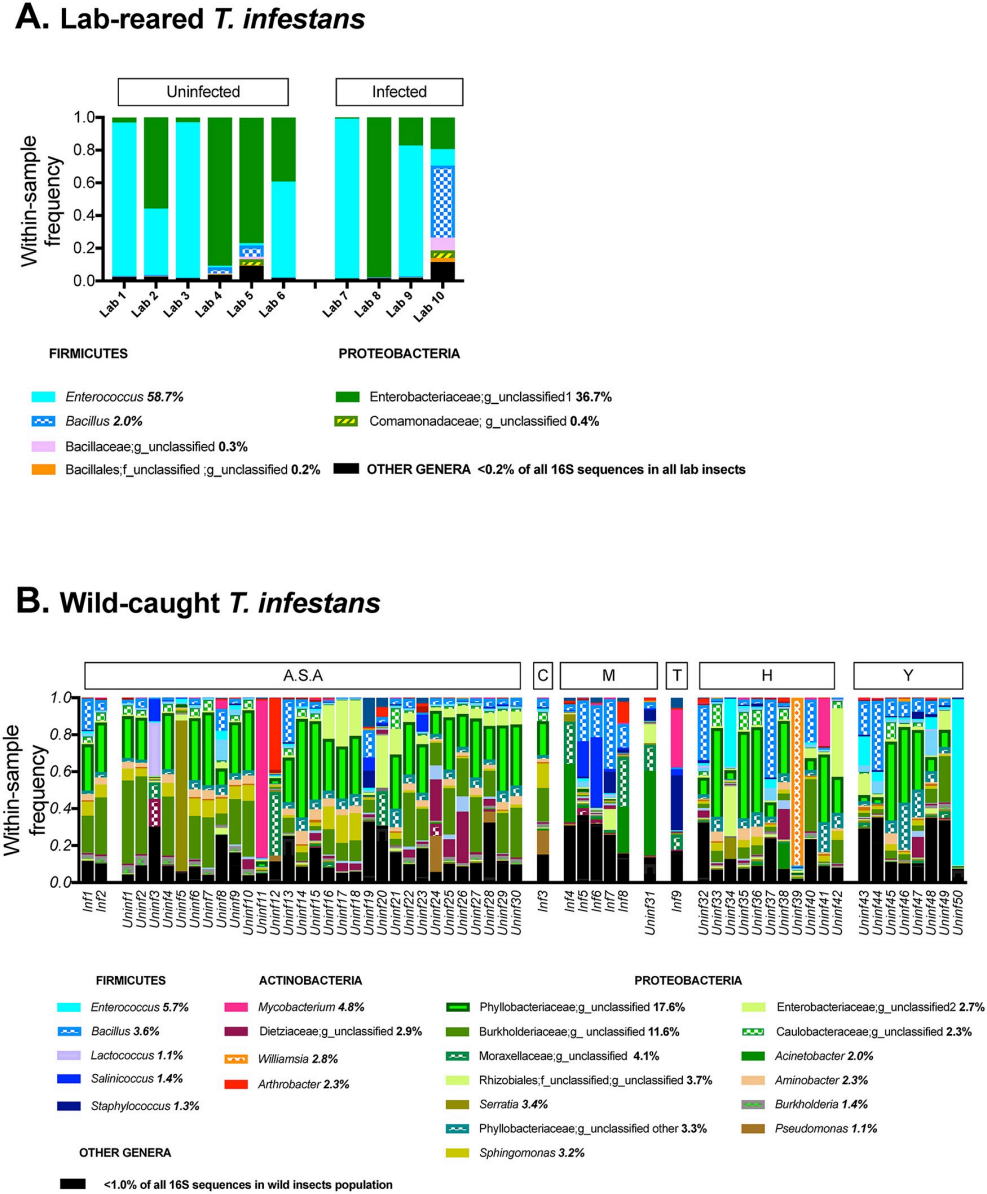
Bacterial genera and their relative abundance in the fecal microbiota of laboratory-reared *T*. *infestans* (A) and wild-caught *T*. *infestans* (B). Each vertical bar represents the microbial composition at the genus level (wherever unambiguous GreenGenes taxonomic classification at the genus level was possible), of each sample, and the within-sample frequency is denoted by the y-axis. **Panel A**. Of 124 genera identified in wild insects, 96 were also found in the lab-reared *T*. *infestans*. Only three genera dominated the overall microbiome of all lab insects: 97.4% of the overall microbiome of lab-reared insects was due to *Enterococcus* (58.7% of all 16S sequences of lab insects), an unclassified *Enterobacteriaceae* genus (36.7%), and *Bacillus* (2.0%). Abbreviations: Inf = *T*. *cruzi*-infected; Uninf = not infected with *T*. *cruzi*; A.S.A = Alto Selva Alegre District; C = Cayma District; M = Murco District; T = Tiabaya District; H = Hunter District; Y = Yura District. **Panel B**. Wild insect samples are ordered by their district of capture and *T*. *cruzi*-infected status. A total of 124 genera were identified for all wild *T*. *infestans*. The 22 genera listed comprised 84.5% of the overall microbiome of wild insects with individual relative abundances ranging from 1.1–17.6% of all 16S sequences in wild insects. The population-level relative abundance of these genera (calculated as the sequence counts assigned to that genera/total sequence counts in all wild insects x 100) is given. The remaining genera, which each made up <1% of all 16S sequences from wild insects, are listed as “Other genera” in the legend.

After demultiplexing and elimination of low-quality reads, 717,470 reads remained. Operational Taxonomic Units (OTUs) were defined using a similarity threshold of 97% followed by taxonomy assignment with respect to the GreenGenes database [40]. After quality filters were applied to exclude potentially spurious OTUs and Cyanobacteria (comprising 0.01% and 0.05% of all reads, respectively), a total of 672,181 reads were retained for downstream analyses of α-diversity, β-diversity, and differential abundance of taxa (mean number of reads per sample = 9,676; range = 673–23,174). Only two samples belonging to wild insects had <1000 sequences and for many of the downstream analyses affected by sample depth, these two samples were excluded. The other samples of wild insects all had >2000 sequences. In the complete dataset of 69 triatomines we identified 32 taxa at the taxonomic rank of order (S2 Table), 70 at the family level (S3 Table), and 124 taxa at the genus level (S3 Table). The genus-level bacterial composition of all lab insects (n = 10) and all wild (n = 59) is shown in Fig 1A and 1B, respectively.

### Microbiota comparison of laboratory-reared *vs*. wild *T*. *infestans*

We first compared the α- and β-diversities of 2013 and 2015 laboratory-reared insects to confirm the validity of combining the data of these insects. The microbial composition of 2013 and 2015 insects did not differ significantly when comparing α-diversity with number of observed taxa as the metric (*p* = 0.430) and β-diversity (weighted Unifrac, ANOSIM R = -0.079, *p* = 0.605) (S1 Fig). For subsequent analyses, 2013 and 2015 bugs were analyzed together.

**Fig 2:**
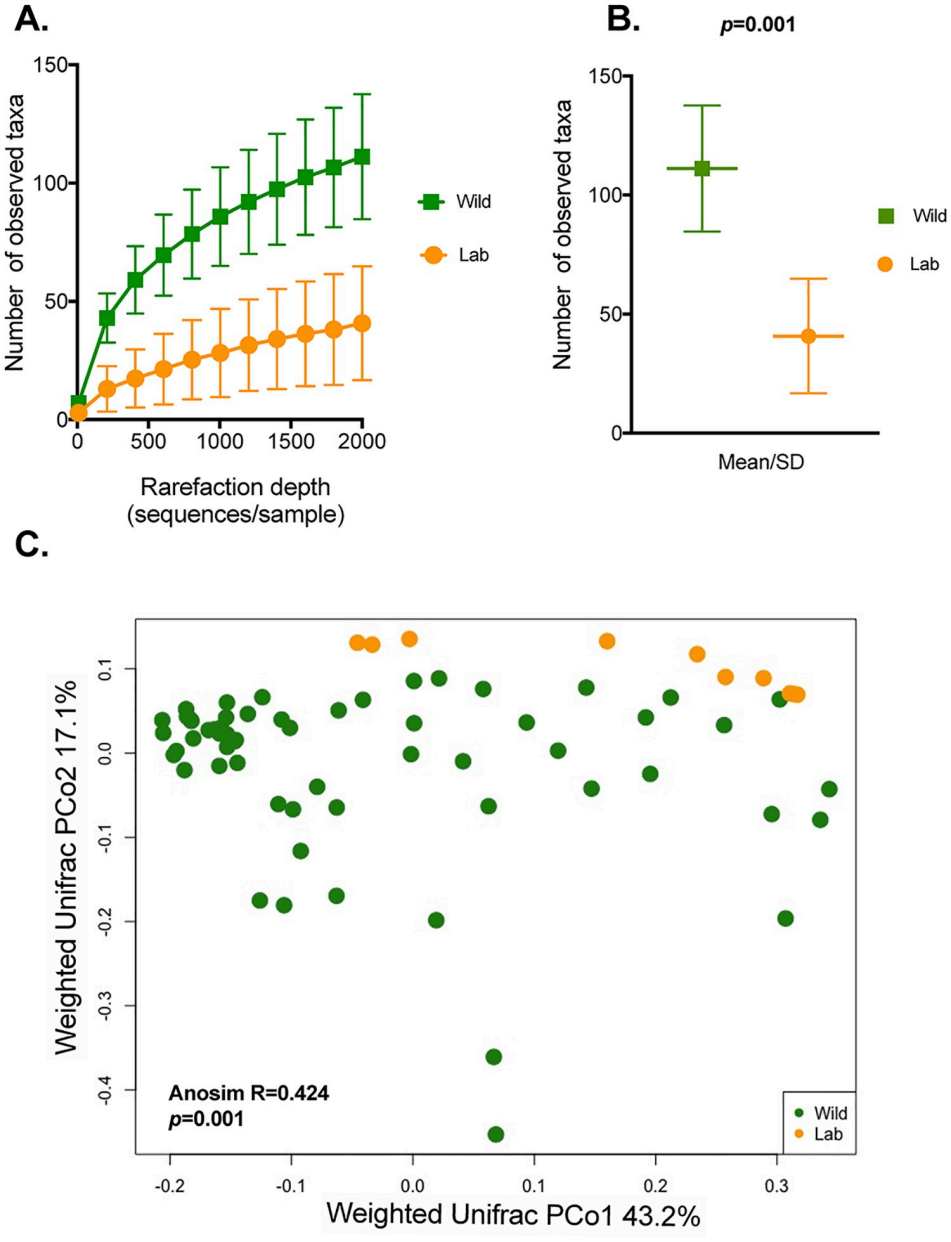
α- and β-diversity of laboratory-reared and wild-caught *T*. *infestans*. Lab insects are shown in orange and wild insects are shown in green. **Panels A and B**. α-diversity was investigated at a rarefaction depth of 2000 sequences per sample with observed number of taxa as the metric (**Panel A**). The number of observed taxa for lab and wild insects was then compared by averaging the iterations of rarefactions within sample groups and using non-parametric two-sample *t*-tests with 1000 Monte Carlo permutations to calculate the *p*-value (**Panel B)**. The origin of insect collection (lab or wild) was significantly associated with the number of taxa identified (p < 0.001). **Panel C**. β-diversity was estimated with weighted Unifrac at a depth of 2000 sequences/sample and tested for significant differences with the non-parametric permutation ANOSIM test. Variation patterns were visualized with principal coordinate analysis (PCoA). The weighted Unifrac variation patterns indicate differences between the bacterial microbiota of lab *vs*. wild insect, as samples from the two groups cluster separately. This was formally supported by non-parametric permutation ANOSIM test which found significant β-diversity clustering between the lab and wild insects with 1000 permutations (R = 0.424, *p* = 0.001).

Next, we compared the microbial composition between wild-caught and laboratory-reared *T*. *infestans*. All of the bacterial taxa identified in the complete dataset were seen in the wild-caught insects. The microbiomes of laboratory-reared *T*. *infestans* were a subset of the wild-caught *T*. *infestans* microbiota (S2 Fig) and included 56 of the total 70 bacterial families (80.0%, S3 Table) and 96 of the total 124 genera identified (77.4%, S3 Table). The 28 genera absent from the lab-reared insects included both rare taxa in the wild insect population (present in only 2–5 of the 59 wild insects) and common taxa (present in up to 40/59 wild insects) (S3 Table) and represented mainly soil-associated bacteria [48]. By rarefaction analyses of number of observed taxa (Fig 2A and 2B), the α-diversity of laboratory-reared *T*. *infestans* was significantly lower than that of wild-caught *T*. *infestans* (*p* = 0.001) at increasing rarefaction depths (2000, 5000 and 10000 sequences, S3 Fig) and by the additional metrics Chao1 and Faith’s Phylogenetic Diversity (S4 Fig).

The greater α-diversity in wild compared to lab insects was evident when inspecting the taxa plots (Fig 1), as only three genera dominated the microbiomes of the lab-reared insects: 97.4% of the overall microbiome of lab-reared insects was composed of *Enterococcus* (58.7%), an unclassified *Enterobacteriaceae* genus (36.7%), and *Bacillus* (2.0%) (Fig 1A), with the remaining 2.6% of sequences assigned to the other 93 genera. The high prevalence of this unclassified *Enterobacteriaceae* genus is notable since it was present in only 0.25% of all 16S sequences of wild *T*. *infestans* (S3 Table), the largest difference in relative abundances observed between wild and lab insects. In contrast to lab insects which were dominated by only three genera only overall, 22 genera were found to be responsible for 84.5% of the overall microbiome among all wild insects (relative abundances among all sequences of wild insects ranging = 1.1–17.6%, Fig 1B); the remaining 14.5% of wild insect sequences were assigned to the other 102 genera observed. *Enterococcus* and *Bacillus*, which had high relative abundances in lab insects, were among the 22 genera in wild insects, present in 5.7% and 3.7% of all 16S sequences of wild insects, respectively (Fig 1B, S3 Table). Not all individual insects were dominated by the same genus and 19 distinct genera were observed to be dominant across individual insects (i.e. having the highest within-sample frequency of all taxa identified in that sample) with frequencies ranging from 11.9%-91.3% and generally reflecting the taxa seen at the population level (Fig 1B). *Serratia*, *Williamsia*, and *Staphylococcus* genera, noted to be important components of triatomine microbiomes in other studies [16, 22, 31], were highly prevalent in only a few samples (Uninf05, Uninf39, and Inf9, Fig 1B). 17.29% of all 16S sequences from wild insects were *Actinomycetales* compared to only 0.18% of all lab insect 16S sequences. *Rhodococcus*, *Dietzia*, and *Corynebacterium*, *Actinomycetes* postulated to be functionally relevant to the microbiome of triatomines [16], were found in the majority of the insects (S3 Table), but at low overall relative frequencies. We identified *Wolbachia* in 51/59 (86.4%) of wild *T*. *infestans* (9/9 *T*. *cruzi*-infected and 42/50 uninfected) and in 8/10 (80.0%) of lab insects (4/4 *T*. *cruzi*-infected and 4/6 uninfected) (S3 Table). *Wolbachia* is a symbiont of many other insect species that act as vectors of human pathogens and has been found to be important for vector fitness and to influence the carriage of human pathogens by these vectors [13–15, 49–54]. In our samples, however, *Wolbachia* was present at very low within-insect frequencies which averaged 0.06% in wild *T*. *infestans* and 0.02% in lab *T*. *infestans*, and they did not change with respect to *T*. *cruzi*-infection.

Differences between wild and lab insects’ microbiota were also reflected in β-diversity analyses, with statistically significant ANOSIM tests and clear separation on PCoA between the wild and lab insect microbiomes by three different methods: weighted Unifrac ANOSIM R = 0.424, *p* = 0.001 (Fig 2B); Bray-Curtis ANOSIM R = 0.796, p = 0.001 (S3C Fig); and unweighted Unifrac ANOSIM R = 0.593, p = 0.001 (S3D Fig). In addition, analysis of differences in relative abundances by DESeq2 showed that almost half of the microbiome common to both wild and lab insects (44.8%, 43/96 genera) was differentially abundant (S5 Fig).

### Month and location of collection are not associated with microbiome composition in wild uninfected *T*. *infestans*

To investigate factors influencing microbiome variation among wild uninfected *T*. *infestans*, we explored the effect of time of collection (month of the year) and of place of collection (Arequipa districts) on the microbial composition by independently analyzing uninfected *T*. *infestans* collected from the Alta Selva Alegre (A.S.A) district only (n = 30) from November 2011 to May 2012 and uninfected *T*. *infestans* collected in March 2012 from three different districts (Yura n = 2, Hunter n = 7, and A.S.A n = 7). Month of insect capture was not associated with microbial composition by α-diversity (observed number of taxa, *p* = 1.000 for all pairwise comparisons) and β-diversity analyses (R = 0.120; *p* = 0.080, S6 Fig). Similarly, when time of collection was restricted to a single month, district of collection had minimal effect on microbial diversity of wild uninfected *T*. *infestans* from Yura, Hunter, and A.S.A districts, both in α-diversity (number of observed taxa, range *p* for all pairwise comparisons = 0.960–1.000) and β-diversity analysis (ANOSIM R = 0.019, p = 0.348, S7 Fig).

### Developmental stage is not associated with microbiome composition in wild *T*. *infestans*

The microbial composition across developmental stage (S8 Fig, Panel A) was not associated with α-diversity (observed number of taxa, *p* = 1.000 among all comparisons, S8 Fig Panels B and C) or β-diversity associated with developmental stage (weighted Unifrac ANOSIM R = -0.042, *p* = 0.767, S8 Fig Panel D). However, small numbers of third and fourth instars may have had limited power to detect differences between older and younger developmental stages.

### *T*. *cruzi* is associated with variation in the microbiota of wild *T*. *infestans*, but not with that of lab-reared *T*. *infestans*

**Fig 3:**
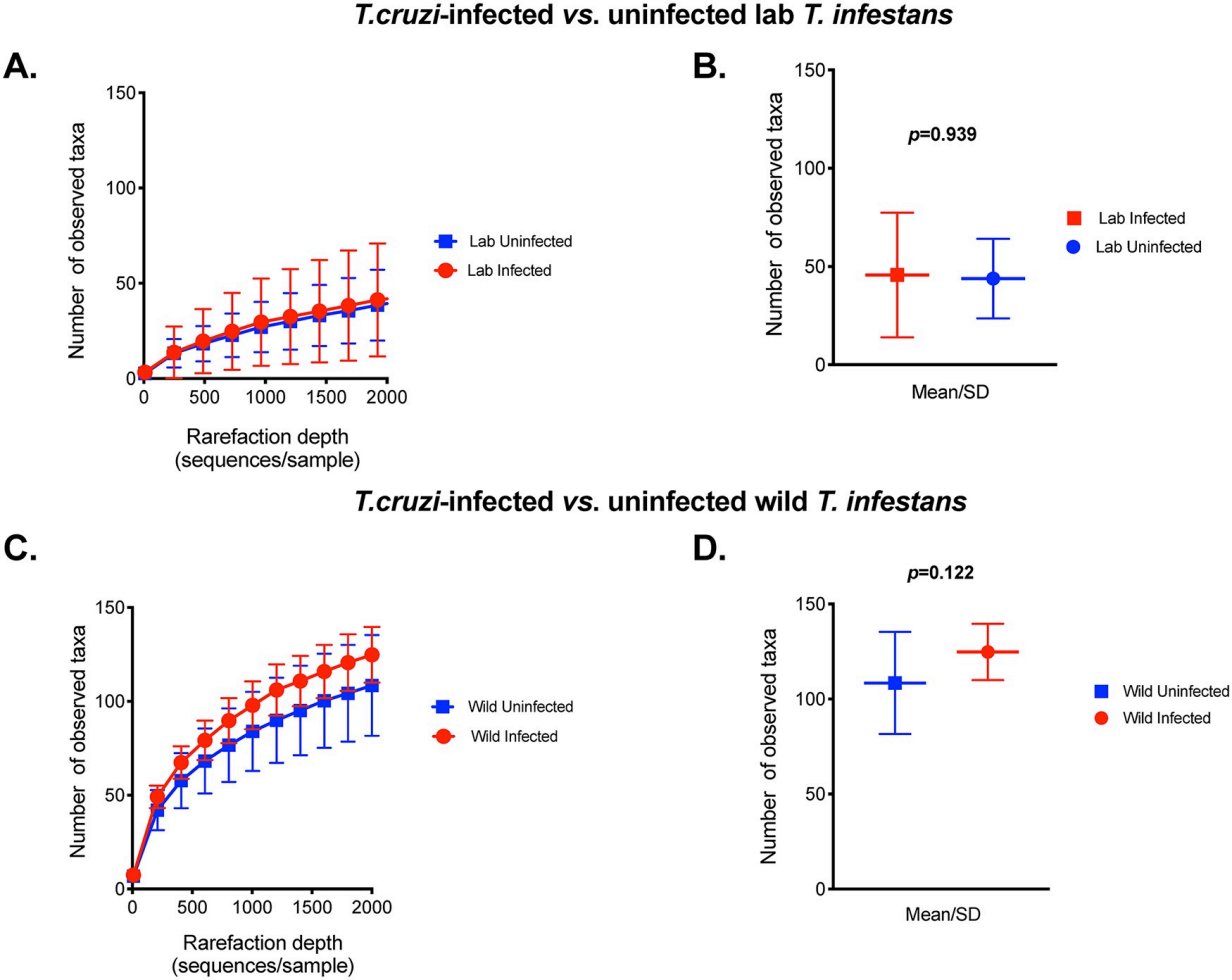
α-diversity of laboratory-reared (Panels A and B) and wild-caught *T*. *infestans* (Panels C and D) with respect to *T*. *cruzi* infection. We investigated α-diversity at a rarefaction depth of 2000 sequences per sample with observed number of taxa as the metric for both lab and wild insect samples. The number of observed taxa for *T*.*cruzi*-infected and uninfected lab and wild insects was then compared by averaging the iterations of rarefactions within sample groups and using non-parametric two-sample *t*-tests with 1000 Monte Carlo permutations to calculate the *p*-value. *T*. *cruzi* infection was not associated with α-diversity and β-diversity of either lab (p = 0.939) or wild insects (0.122).

**Fig 4:**
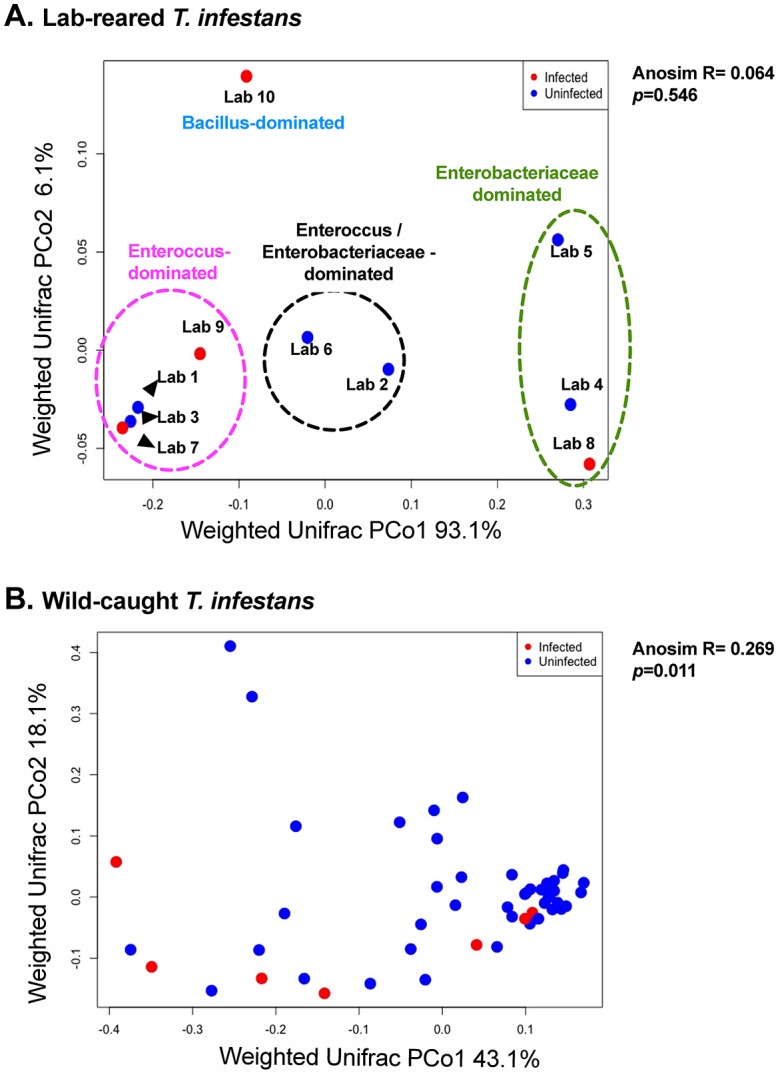
β-diversity of laboratory-reared (Panel A) and wild-caught *T*. *infestans* (Panel B) with respect to *T*. *cruzi* infection. β-diversity analysis was calculated with weighted Unifrac as the metric and visualized with principal coordinate analysis (PCoA). β-diversity clustering between *T*.*cruzi*-infected and uninfected lab and wild insects was assessed with non-parametric permutation ANOSIM tests with 1000 permutations. **Panel A**: The weighted Unifrac results indicated that β-diversity was not associated with *T*. *cruzi* infection for lab-reared insects (R = 0.064, *p* = 0.546). Rather, differences among lab insects were better explained by the dominant within-sample genus. Samples dominant by *Enterococcus* are circled in pink, those dominated by *Enterobacteriaceae* are circled in green, those dominated by both *Enterococcus* and *Enterobacteriacea* in black, and the one sample dominated by *Bacillus* in blue. **Panel B**: The ANOSIM test of the weighted Unifrac analysis suggested significant differences between infected and uninfected wild insects (R = 0.269, *p* = 0.011), despite visualization by PCoA not showing a clear separation.

We examined whether *T*. *cruzi* infection is associated with the microbiota composition of lab-reared and wild-caught *T*. *infestans*. Four of the 10 lab insects and 9 of the 59 wild-caught *T*. *infestans* were *T*. *cruzi*-infected. *T*. *cruzi* infection did not affect α-diversity of either lab or wild insects by any metric (number of observed taxa Fig 3; Chao1 and Faith’s PD, S9A–S9D Fig). Next, we estimated β-diversity between *T*. *cruzi*-infected and uninfected lab insects by weighted Unifrac, tested the differences in variation by the ANOSIM test and the variation patterns were visualized by PCoA. β-diversity was not associated with *T*. *cruzi* infection in lab insects by weighted Unifrac (ANOSIM R = 0.064, *p* = 0.546, Fig 4A); this was also the case when unweighted and Bray-Curtis methods of β-diversity analysis were used (S10 Fig). Variation in microbiome of lab-reared insects was better explained by the dominant bacterial genus within each sample (Fig 4A). In wild insects, on the other hand, *T*. *cruzi* infection was associated with variation in β-diversity by weighted Unifrac analysis (ANOSIM R = 0.269; *p* = 0.011, Fig 4B) as well as unweighted Unifrac (R = 0.215, *p* = 0.012, S11 Fig) and Bray-Curtis methods (R = 0.250, p = 0.012, S11 Fig). The variation patterns were visualized by PCoA (Fig 4B, S11 Fig).

### Differential abundance analysis in *T. cruzi*-infected and uninfected wild *T. infestans*

**Fig 5:**
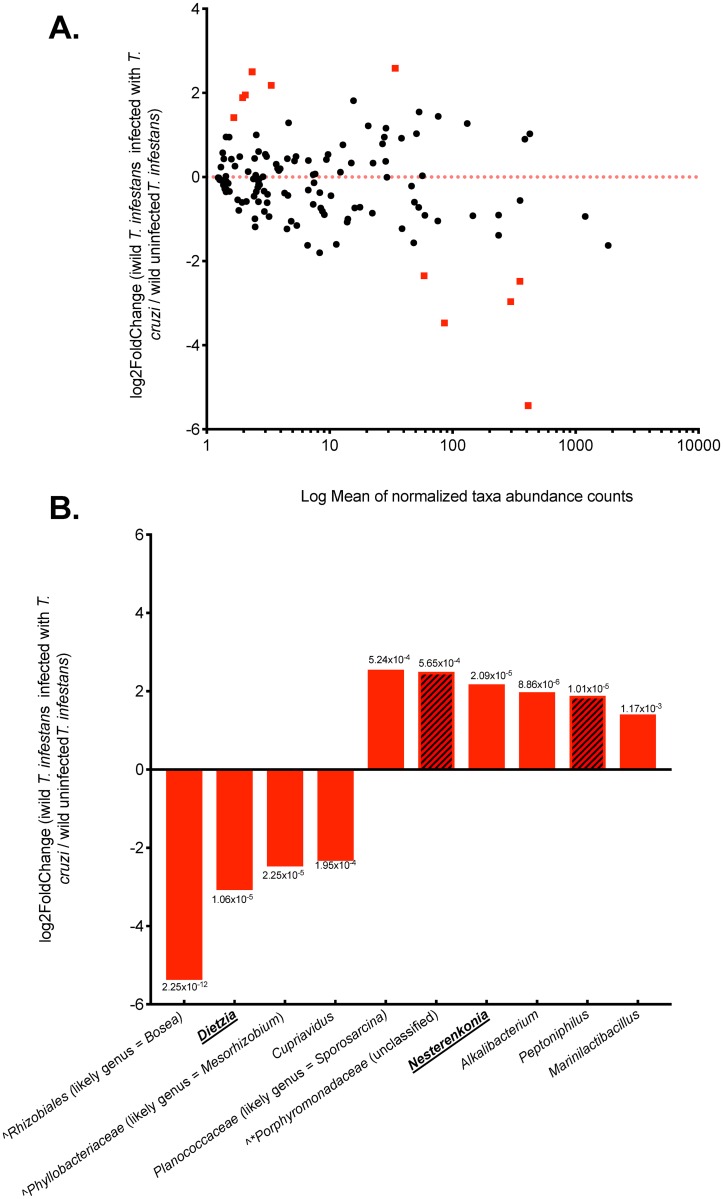
Differential abundance analysis by the DESeq2 method [47] comparing the relative abundances of taxa identified in wild *T*. *infestans* infected with *T*. *cruzi* vs. uninfected *T*. *infestans*. **Panel A. MA-plot (an application of Bland-Altman plot) showing the log2 fold difference in relative taxon abundance when comparing taxa of *T*. *cruzi*-infected wild insects to taxa of uninfected wild insects**. Each point represents one of the 124 taxa identified in wild insects overall. Points above the line (positive differences in relative abundance) depict taxa which were overrepresented in infected wild insects compared to uninfected wild insects, and those below the line (negative differences in relative abundance) depict taxa which were underrepresented in infected insects compared to uninfected insects. Ten genera were identified to have statistically significant differences in their relative abundance between infected and uninfected wild insects (*p* <0.01) and their points are colored red. **Panel B. DESeq2 analysis identified 10 bacterial taxa that were differentially abundant between *T*. *cruzi*-infected and uninfected wild insects**. OTU sequences for which genus identification was not possible by consulting taxonomies in the GreenGenes database (i.e. *Rhizobiales*, *Phyllobacteriaceae*, *and Porphyromonadaceae)* were aligned with BLAST against NCBI’s GenBank 16S sequences and the likeliest taxonomic lineage is given in parentheses. These 10 genera had Bonferroni-corrected *p*-value ranging from 2.25x10^-12^–0.01, and the p-values are given above each bar. Bars above the line (positive differences in relative abundance) depict taxa which were overrepresented in infected wild insects compared to uninfected wild insects, and those below the line (negative differences in relative abundance) depict taxa which were underrepresented in infected insects compared to uninfected insects. Taxa denoted by ^ were identified by Spearman’s Rho with bootstrapping and 100 permutations to be associated with *T*. *cruzi* infection; shaded taxa were associated with increased odds of *T*. *cruzi*-infection by Fisher’s exact test (*Peptinophilus*, OR = 9.17; 95% CI = 1.44, 58.15; unspecified *Porphyromonadaceae* genus, OR = 18.00; 95% CI = 2.62, 136.55). Only the unspecified *Porphyromonadaceae* genus was also identified by Kruskal-Wallis non-parametric statistic, Bonferroni-corrected *p*<0.005). Taxa which belong to the *Actinomycetes* group are underlined.

Ten bacterial genera were found to have relative abundances which were significantly different with respect to *T*. *cruzi* infection in wild insects by DESeq2 analysis (Fig 5A and 5B, *p*<0.01). Using the GreenGenes database, these 10 genera were identified as an unclassified genus and family of the order *Rhizobiales*, *Dietzia*, *Alkalibacterium*, *Peptoniphilus*, *Nesterenkonia*, *Cupriavidus*, *Marinilactibacillus*, and three unclassified genera of the *Porphyromonadaceae*, *Phyllobacteriaceae*, and *Planococcaceae* families. *Porphyromonadaceae* was also identified as being associated with *T*. *cruzi*-infected insects by all other association tests conducted: Kruskal-Wallis non-parametric statistic (Bonferroni-corrected *p*<0.005), Spearman’s Rho (*p*<0.001, with bootstrapping and 100 permutations) and Fisher’s exact test (OR 18.00; 95% CI = 2.62–136.55). The unclassified *Rhizobiales* and *Phyllobacteriaceae* (*p*<0.001, Spearman’s Rho with bootstrapping and 100 permutations) and *Peptinophilus* (OR 9.17; 95% CI = 1.44–58.15 by Fisher’s exact test) were also associated with increased odds of *T*. *cruzi* infection.

For the *Rhizobiales*, *Phyllobacteriaceae*, *Porphyromonadaceae*, and *Planococcaceae* GreenGenes assignment was ambiguous at the genus level and thus we consulted the 16S sequences deposited in the NCBI repository GenBank by BLAST [43]. The likeliest taxonomic lineages for *Rhizobiales*, *Phyllobacteriaceae*, and *Planococcaceae* sequences are the genera *Bosea* (sequence identity range = 98.65–100.00%; e = 0; query cover = 100.00%), *Mesorhizobium* (sequence identity range = 97.91–100.00%; e = 0; query cover = 100.00%), and *Sporosarcina* (sequence identity range = 97.89–100.00%; e = 0; query cover = 100.00%), respectively. Taxonomically, *Phyllobacteriaceae* are part of the *Rhizobiales* order [48]. GenBank sequences producing significant BLAST alignments with our *Porphyromonadaceae* sequences were all uncultured *Porphyromonadaceae* bacteria.

## Discussion

The microbial communities colonizing arthropod vectors of human pathogens influence the ability of pathogens to survive, reproduce and transmit to humans and other hosts [5–8]. Microbiota-based strategies to control and prevent vector borne illnesses have now garnered more attention, with efforts to curb dengue transmission using *Wolbachia*-based microbiota modifications of *Aedes* mosquitoes being the most advanced to date [13, 15, 51]. Such strategies are also being explored for triatomines, the insect vectors of Chagas disease. However, microbiome characterizations of triatomines remain relatively limited, particularly for *T*. *infestans*, the principal vector in the Southern Cone of South America. Our work describes, for the first time, the hindgut microbiota of *T*. *infestans* with sensitive high-throughput approaches in both laboratory-reared and wild-caught *T*. *infestans* and investigates the relationship between *T*. *cruzi* infection and microbiota profiles.

The existing triatomine microbiota literature suggests that the largest differences in microbial composition are driven by triatomine species [20, 23, 24, 26–28]. Indeed, we found differences between our *T*. *infestans* microbiota and that of other species’ microbiota, but also many similarities. We also observed notable differences between our *T*. *infestans* microbiota and that of *T*. *infestans* reported in other studies. We observed that *Enterobacteriales* and *Bacillales*, previously shown to dominate the microbiota of *Rhodnius prolixus* [28] and *T*. *dimidiata* [20], respectively, but not *T*. *infestans* [25], occurred at high relative abundances in both our lab (*Enterobacteriales*, 37.0%; *Bacillales* 2.5%) and wild insect populations (*Enterobacteriales*, 7.6%; *Bacillales* 9.0%). *Enterococcus* and *Enterobacteriaceae*, which were found at high relative abundance in our lab-reared *T*. *infestans*, have also been found to be significant microbial components of lab-reared *R*. *prolixus*, *T*. *protracta*, *T*. *recurva*, and *T*. *vitticeps* [27]. When comparing our findings to the other reports of *T*. *infestans* microbiota [16, 22, 25, 31], we found that *Clostridiales* and *Rhodocyclales* were less prevalent in our study.

Some genera which have been previously identified as important members of the microbial communities of triatomines, including of *T*. *infestans*, were absent from our insects (*Arsenophonus)*, observed in the majority of both lab and wild bugs but at low relative abundance (*Rhodococcus* and *Corynebacterium)*, or found at high relative abundance in only a few wild *T*. *cruzi*-uninfected samples (*Mycobacterium*, *Williamsia*, *Serratia*). *Arsenophonus* and *Corynebacterium* are candidates for paratransgenic techniques to prevent. *T*. *cruzi* infection in *T*. *infestans* [25, 55]; *Rhodococcus* has been already established in molecular strategies for *T*. *cruzi* control in another triatomine vector of Chagas, namely *R*. *prolixus* [16, 22, 55]; and the *Serratia* genus contains members shown in *R*. *prolixus* to have trypanolytic activity on specific *T*. *cruzi* strains [31]. Finally, the *Wolbachia* genus was detected in the majority of our wild and lab-reared triatomines but at low overall relative abundance (0.05% of all 16S sequences from wild insects and 0.02% of all 16S sequences from lab insects). This low overall relative abundance can also be due to our rectal sampling, because it has been shown that triatomine intestinal microbes are typically present in low numbers in the rectum [17] where *T*. *cruzi* undergoes development [21]. The *Wolbachia* genus contains members which are common symbionts in many insect species but best described in mosquito vectors of malaria, dengue, chikungunya [14, 49–51, 53, 56]. *Wolbachia* has been demonstrated to affect vector survival [51, 53, 56] and the human pathogen as well [13, 14, 49–54, 56]. Previously, *Wolbachia* has only been observed in various organs of *Rhodnius pallescens* [57], but not in any other triatomine species [23, 24, 26, 28, 57]. Whilst we cannot establish based on our data that *Wolbachia spp*. are true symbionts of *T*. *infestans*, the fact that we observe this genus in 51/59 of wild insects and 8/10 of lab insects is indicative of symbiosis. Further efforts are needed to better understand whether *Wolbachia* and the other bacteria we found in the majority of both our lab and wild bugs, but at low relative abundance in the hindgut, are rare microbes or present at higher densities in other intestinal segments and what functional role, if any, they may play in the *T*. *infestans* lifecyle and *T*. *cruzi* infection. Recent studies of human and plant microbiomes have shown that even rare gut microbes can be implicated in key processes, such as preventing pathogen colonization and boosting host immunity [58–61]. Therefore, rare microbes may be equally as relevant in shaping the structure of the microbiome as the taxa with high relative abundance taxa and should not be discounted from characterizing a microbial ecosystem [62].

Microbiota varied substantially between our lab and wild insects, particularly with respect to environmental species and *Actinomycetes*. All known triatomine symbionts are members of the *Actinomycetes* [18] and *Actinomycetes* are the main producers of naturally-occurring antibiotics [63]. As in previous studies [23, 24] we observed that the overall diversity of lab *T*. *infestans* was reduced compared to wild *T*. *infestans*. The missing microbes in lab *vs*. wild insects pertained primarily to soil-associated bacteria (such as *Rhizobiales* and *Burkholderiales*). Even among the 96 genera which were common to both lab and wild bugs, we found significant differences: only three genera (*Enterococcus*, an unidentified genus of *Enterobacteriaceae*, and *Bacillus*) dominated the lab insects, whereas 22 distinct genera dominated the overall microbiota of the wild *T*. *infestans*. Whilst we detected *Actinomycetes* in all lab and all wild insects we sampled, their overall diversity and relative abundance was significantly reduced in lab *vs*. wild insects: 17.3% of all 16S sequences from wild insects were *Actinomycetales* (representing 30 distinct genera) compared to only 0.18% of all lab insect 16S sequences (representing 22 distinct genera). Higher microbial complexity in field triatomines due to soil-associated bacteria has been previously reported for *T*. *brasiliensis* and *T*. *pseudomaculata* [24]. This suggests that environmentally-acquired microbes by the wild insects while off-host/ host-seeking is an important source of these differences. The low relative abundance of *Actinomycetes* symbionts in our lab insects can be due to rectal sampling or to our rearing protocol (i.e. indirect coprophagy, whereby as third instars our lab insects fed on chickens on which adult triatomines previously fed on). The latter may not have seeded the full collection of gut symbionts. These differences reflect the overall decreased diversity of laboratory-reared triatomines and highlight that the microbiome of insectary-reared *T*. *infestans* may not represent that of wild populations, even in the same geographic area.

The effect of environmentally-acquired bacteria and *Actinomycetes* symbionts on *T*. *cruzi* infection of wild *T*. *infestans* is not well characterized. *Actinomycetes* symbionts in triatomines have been shown to influence insect gut homeostasis by providing useful metabolites [18]. In addition, it has been postulated that acquisition of important *Actinomycetes* symbionts is in fact dependent on the environment [64], because of coprophagy from soil contaminated with feces [16]. Numerous examples of the relevance of environmentally-acquired extracellular microbes to other insects’ fitness exist (reviewed here [65]). For instance, it has been demonstrated that lab-hatched *Ixodes scapularis* ticks lacking the environmentally-acquired microbes carried a larger proportion of human pathogenic *Rickettsia spp*. than field-caught ticks, indicating that environmentally-acquired symbionts outcompete *Rickettsia* [66]. Our data suggest that environmentally-acquired species and/or *Actinomycetes* may be important to *T*. *cruzi* infections because of two observations. First, we found differences between *T*. *cruzi*-infected and uninfected wild insects and 8/10 genera differentially abundant between infected and uninfected bugs were environmental microbes, primarily found in soil, or *Actinomycetes* (*Bosea*, *Mesorhizobium*, *Dietzia*, and *Cupriavidus* were underrepresented in infected bugs; *Sporosarcina*, *Nestenrenkonia*, *Alkalibacterium*, *Marinilactibacillus* were overrepresented in infected bugs). Second, the microbiome structure of our lab insects which were deficient in both environmental species and *Actinomycetes* was not associated with *T*. *cruzi* infection.

Little is known about the relevance of the 10 different genera which differed significantly in their relative abundance between infected and uninfected wild bugs to *T*. *infestans* or *T*. *cruzi*. *Dietzia* is of particular interest because it has appeared in the triatomine microbiome literature [24–27, 29, 67] and it is an *Actinomycetes* [68]. In our sample, the *Dietzia* genus was underrepresented in infected *vs*. uninfected wild triatomines. *Dietzia* is a relatively newly described genus of the *Actinomycetales* order, suborder *Corynebacterineae*. In the past it has been misclassified as *Rhodococcus spp*. because of their striking similarity in Gram morphology and colony presentation [48]. Interestingly, genetically-modified *Rhodococcus rhodnii* expressing anti-trypanocidal agents in the triatomine gut was the first ever microbiota-based strategy of vector control [16]. *Dietzia spp*. have been described as symbionts of other insect vectors of human pathogens, including the tsetse fly *Glossina pallidipes*, the vector of African trypanosomiasis [69], and of the myaisis-causing parasitic fly *Wohlfahrtia magnifica* [70]. Because *Dietzia* is postulated to contain species which may produce antimicrobials [68], its metabolic richness in relation to *T*. *infestans* and *T*. *cruzi* cannot be overlooked, particularly in the context of widespread insect-*Actinomycetes* symbioses [71], where insects gain essential metabolites (such as B-complex vitamins in triatomines [18]) or are known to actively recruit antibiotic-producing *Actinobacteria* for protection [72].

Like our study, previous studies of triatomine microbiomes and their relationship to *T*. *cruzi* infection have also found differences between infected and uninfected bugs, but these findings do not always agree: Castro *et al*. [73] reported that resident microbiota of *R*. *prolixus* may create a hostile environment for the parasite, as pre-treatment with antibiotics in the blood meal increased *T*. *cruzi* infectivity ten-fold; however, *in vitro* assays suggested that the parasites inhibited bacterial growth as well [73]. In contrast, other studies [25] found that lab-reared triatomines challenged or infected with *T*. *cruzi* had higher α-diversity. We did not find that α-diversity differed by infection status in either lab or wild bugs.

Although we found associations between microbiota and *T*. *cruzi* infection in wild insects and found *Dietzia* to be a potentially important taxon to *T*. *cruzi* infection, we cannot infer the directionality of these relationships. Thus, we cannot conclude whether these differences in microbiome structure make the bugs susceptible to *T*. *cruzi* infection or whether *T*. *cruzi* itself changes the microbiome. To parse out the effect of microbial composition on triatomine susceptibility to *T*. *cruzi* infection, experiments that sample lab-reared insects before and after *T*. *cruzi* infection are needed. Work examining the effect of *Rhodococcus* on lab-reared *R*. *prolixus* microbiota suggested that the parasite does influence the bug’s microbiome [73]; however, other studies that have investigated the relationship between vector, *T*. *cruzi* and symbionts have suggested that the symbionts may affect *T*. *cruzi* presence and not the other way around, since *T*. *cruzi* has been shown to minimally affect the development and mortality of *T*. *infestans*, as long as the vector has an adequate food supply (reviewed here [18, 30]). This may be because whilst *T*. *cruzi* appears to compete for nutritional resources [30], as long as the insects are not starved, these effects are negligible [30]. However, since regular meals cannot always be guaranteed in the wild, interactions between triatomines’ intestinal environment and *T*. *cruzi* may be quite different. We lacked data of potentially important variables in wild-caught insects, such as length of time since last feeding, blood meal source and duration of *T*. *cruzi* infection to assess these interactions in our wild bug population.

We found significant microbiome variation among individual wild insects. We did not find evidence that seasonality, geography or developmental stage explain this variation. Whilst our findings are in line with a previous report of wild-caught *T*. *protracta* that geography does not explain microbiome variability [27], our collections were spatially limited in relation to the *T*. *infestans* geographic range and our low sample sizes for some districts may have had limited power for spatial analyses. Thus, studies of the microbial structure of wild triatomines across a wider geographic area are needed. This is particularly relevant given our data and those of other studies indicating the importance of environmentally-acquired microbes to the microbiome of triatomines [16, 25]. Our lack of microbiome changes along developmental stages is not in line with other reports of field *T*. *sordida* [29] and lab-reared *R*. *prolixus* [27] which found that the microbiome changes from development stage to the next. This calls for more investigations exploring how the microbiome develops with ontogeny, particularly when considering that all triatomine instars have the ability to transmit *T*. *cruzi*. Finally, a systematic temporal collection of wild triatomines was hindered by low infestation and infection rates at the time in Arequipa and seasonality should not be discounted as an important factor influencing the microbiota of triatomines.

Our study had other limitations as well. It is unclear whether the lack of microbiota differences we observed between infected and uninfected lab insects is due to low sample size or due to our methodology of indirect coprophagy. Indirect coprophagy may not have seeded the lab nymphs with the full diversity of gut symbionts or with high enough numbers to establish gut colonization. We do not believe our laboratory insects to be aposymbiotic because we identified *Actinomycetes* in all lab insects. Finally, because eukaryotic sequencing was beyond the scope of this study we do not have data on the relationship between *T*. *cruzi* and other eukaryotes. Little is known about the eukaryotic microbiome of triatomines, but a single study testing pathogenic fungi as a biopesticide against *R*. *prolixus* found that *T*. *cruzi* infection rendered bugs more resistant to killing by the fungus [50]. The mycome and potentially other parasites undoubtedly have important influences on triatomine biology and immunology as well.

To realize the potential of microbiome-based interventions to decrease the transmission of *T*. *cruzi* and burden of Chagas disease, further studies of vector-gut microbe-*T*. *cruzi* interactions are necessary. To enable a better understanding of the real significance of *T*. *cruzi* on its vectors, such studies of wild triatomines would need to include not only microbiome surveys (including specimens from multiple species, developmental stages and geographic locations), but also metagenomic studies, similar to the study conducted by Carels *et al*. (2017) on lab-reared triatomines which identified major metabolic pathways of gut microbes [67]. Further microbiome and metagenomic characterizations will be particularly important when exploring what role, if any, demonstrated gut symbionts or putative symbionts, such as *Dietzia* or *Wolbachia*, play in *T*. *cruzi-*infection. Additionally, the field will need insectary-based studies of triatomine microbiome before and after *T*. *cruzi* infection to identify any pre-existing bacterial species that are protective, necessary, or encouraging to infection, with the caveat that there are large differences between laboratory-reared and wild bugs’ intestinal flora. These data are necessary to set up real-world trials of interventions that modify the bugs’ microbiomes to make them hostile to *T*. *cruzi* infection.

## Supporting information

S1 Table Baseline characteristics of *T*. *infestans* samples in this study. (DOCX)

S2 Table Bacterial taxa identified at the taxonomic level of order. In the complete dataset of 69 triatomines we identified 32 taxa at the taxonomic rank of order.
(XLSX)

S3 Table Bacterial taxa identified at the taxonomic level of family and genus and their prevalence among wild (infected and uninfected) and lab insects (infected and uninfected). (XLSX)

S1 Fig Comparison of α- and β-diversities of 2013 and 2015 laboratory-reared insects to validate that the data of these insects can be analyzed as part of the same sample. Panel A and B. The microbial composition of 2013 and 2015 insects did not differ significantly when comparing α-diversity with number of observed taxa as the metric (p = 0.430). Panel C. β-diversity (weighted Unifrac, ANOSIM R = -0.079, p = 0.605) did not differ significantly between 2013 and 2015 laboratory-reared insects. For subsequent analyses, 2013 and 2015 bugs were analyzed together.
(TIF)

S2 Fig Overlap of genus-level taxa observed in wild-caught and lab-reared *T*. *infestans*. A total of 124 genera were identified in the complete dataset. All of the 124 genera were seen in the wild-caught *T*. *infestans*. Of these 124 genera, 96 of these were also seen in laboratory-reared *T*. *infestans*.
(TIF)

S3 Fig α-diversity, by observed number of taxa, of wild-caught and lab-reared *T*. *infestans* at different rarefaction depths of 2000 sequences (A), 5000 sequences (B), and 10000 sequences (C). The number of observed taxa in lab insects was consistently lower than that in wild-caught insects (*p*<0.001), irrespective of sequence depth, indicating that a sequence depth of 2000 sequences for α-diversity analyses is adequate.
(TIF)

S4 Fig α-diversity (Chao1 and Faith’s Phylogenetic Diversity) and β-diversity (Bray-Curtis and Unweighted Unifrac) of wild-caught and laboratory-reared *T*. *infestans*. Wild insects are shown in green and lab insects are shown in orange. **Panels A and B**. α-diversity (Panel A) of laboratory-reared and wild-caught *T*. *infestans* was investigated at a depth of 2000 sequences/samples with Chao1 and Faith’s Phylogenetic Diversity (PD) as metrics. The α-diversity of wild and lab insects’ microbiota was then compared by averaging the iterations of rarefactions within sample group and then using non-parametric two-sample *t*-tests with Monte Carlo permutations to calculate the *p*-values (Panel B). **Panels C and D**. β-diversity of laboratory-reared and wild-caught *T*. *infestans* was computed by Bray-Curtis (Panel C) and Unweighted Unifrac (Panel D) and visualized by PCoA. Bray-Curtis, an abundance-based analysis, indicates separation between wild and lab microbial composition which may be due to differentially abundant taxa which exist between the two groups (refer to S5 Fig). Occurrence-based analysis Unweighted Unifrac also show separation between the wild and lab-reared insects because the microbial composition of lab bugs is a subset of that of wild bugs. These results of the Bray-Curtis and Unweighted Unifrac analyses are by non-parametric permutation ANOSIM tests which found significant β-diversity clustering between the lab and wild insects, with 1000 permutations (Bray-Curtis R = 0.796, *p*<0.001; Unweighted Unifrac R = 0.593, p<0.001).
(TIF)

S5 Fig Differential abundance analysis by the DESeq2 method comparing the relative abundances of taxa identified in laboratory-reared insects compared to relative abundances of taxa identified in wild-caught *T*. *infestans*. **A. MA-plot (an application of Bland-Altman plot)** showing the difference in relative abundance of taxa between lab and wild insects (log2 fold differences). Each point represents one of the 96 taxa common to both the lab and wild insects. Above the line (positive values of abundance differences) depict taxa which were overrepresented in laboratory insects compared to wild insects, and values below the line (negative differences in relative abundances) depict taxa which were underrepresented in lab insects compared to wild insects. Points are colored red if the adjusted *p* value is less than 0.01. A total of 43 of the 96 genus-level taxa common to both lab and wild insects were found to be differentially abundant (i.e. log2 fold change departing from 0) with p<0.01. **B. The genus-level taxonomic classification of 43 of 96 taxa common to both lab and wild insects which were differentially abundant in the above analysis**. The log2 fold difference in relative abundances in lab compared to wild insects is given on the y-axis. Taxa are ordered in increasing *p*-value from left to right (range = 2.392−45–0.009). Similarly to the above graph, points above the line (positive differences in relative abundances) depict taxa which were overrepresented in laboratory insects compared to wild insects, and values below the line (negative differences in relative abundances) depict taxa which were underrepresented in lab insects compared to wild insects.
(TIF)

S6 Fig Temporal associations with microbiome composition of wild *T*. *infestans*. A total of 30 uninfected *T*. *infestans* were collected from A.S.A (Alto Selva Alegre) District across different months from November 2011 to May 2012. **Panels A and B**. The total number of observed taxa did not differ among insects collected during different months (*p* = 1.000 for all pairwise comparisons of observed taxa). There was only one insect captured during the month of April, and this sample was excluded from α-diversity analyses. **Panels C and D**. Heterogeneity in taxonomic composition was observed among insects collected during the same month, which can be gleaned from the bar plots of relative abundance (C) and in weighted Unifrac analysis and PCoA (D). Overall, month of collection was not a driver of diversity in the uninfected *T*. *infestans* collected from A.S.A. District.
(TIF)

S7 Fig Spatial associations with microbiome composition of wild *T*. *infestans*. In the same month of March 2012, a total of 16 uninfected wild insects were collected from three districts (Alto Selva Alegre, A.S.A n = 7; Hunter n = 7; and Yura n = 2). **Panels A and B**. The α-diversity did not differ by district of capture when total number of observed OTUs was interrogated (range = 0.960–1.000 for the pairwise comparisons). **Panels C and D**. β-diversity analyses (weighted Unifrac and visualization by PCoA) also showed that district of capture was not a driver of the variation among insects and heterogeneity within the district was at times a stronger factor contributing in driving this variation, as was the case for sample Uninf20 and Uninf39.
(TIF)

S8 Fig Microbial composition, α-diversity and β-diversity of wild *T*. *infestans* by developmental stage. **Panel A. Taxa plots of wild *T*. *infestans* sorted by developmental stage**. Each vertical bar represents the microbial composition at the genus level (wherever unambiguous GreenGenes taxonomic classification at the genus level was possible), of each sample, and the within-sample frequency is denoted by the y-axis. **Panels B and C. α-diversity by number of observed taxa of wild *T*. *infestans* third instars, fourth instars, fifth instars and adults**. Panel B shows the rarefaction curves of the observed number of taxa of each insect group at a depth of 2,000 sequences and Panel C shows the comparisons of α-diversity between each insect group (by averaging the iterations of rarefactions within sample group and then using non-parametric two-sample *t*-tests with Monte Carlo permutations to calculate the *p*-values). Developmental stage did not associate with α-diversity measures (*p* = 1.000 among all comparisons). **Panel D. β-diversity (weighted Unifrac) of wild *T*. *infestans*, by developmental stage**. β-diversity was estimated with weighted Unifrac and formally tested for differences among insects of different developmental stages using the ANOSIM test. Variations patterns were visualized with PCoA. Developmental stage did not associate with variations in β-diversity of wild T. infestans (ANOSIM = -0.042, *p* = 0.767).
(TIF)

S9 Fig α-diversity by Chao1 and Faith’s Phylogenetic Diversity (PD) of lab and wild *T*. *cruzi*-infected and uninfected *T*. *infestans*. The presence of *T*. *cruzi* infection was confirmed by qPCR and microscopy in both lab and wild insects. Infected insects are shown in red and uninfected insects are shown in blue. **Panels A and B. Lab-reared *T*. *infestans***. α-diversity was investigated at a depth of 2000 sequences/samples with Chao1 and Faith’s Phylogenetic Diversity (PD) as metrics (Panel A). The α-diversity of *T*. *cruzi*-infected and uninfected lab insects’ microbiota was then compared by averaging the iterations of rarefactions within sample group and then using non-parametric two-sample *t*-tests with Monte Carlo permutations to calculate the *p*-values (Panel B). α-diversity did not associate with *T*. *cruzi* infection in lab-reared insects (Chao1 *p* = 0.865, PD *p* = 0.913). **Panels C and D. Wild-caught *T*. *infestans***. As for lab insects, α-diversity was investigated at a depth of 2000 sequences/samples (Panel C), then compared by averaging the iterations of rarefactions within sample group and non-parametric two-sample *t*-tests with Monte Carlo permutations to calculate the *p*-values (Panel D). In wild insects, α-diversity did not associate with *T*. *cruzi* infection (Chao1 *p* = 0.122, PD *p* = 0.138).
(TIF)

S10 Fig β-diversity analyses of laboratory-reared insects infected with *T*. *cruzi* (n = 4, red) and uninfected insects (n = 6, blue). β-diversity was computed by Bray-Curtis (Panel A) and Unweighted Unifrac (Panel B) and visualized by PCoA. The results do not indicate that the microbiotas of infected and uninfected lab-reared insects are significantly different. This finding is supported by non-parametric permutation ANOSIM tests of the Bray-Curtis and the Unifrac β-diversity, which did not find significant β-diversity clustering between the infected and uninfected insects, with 1000 permutations (Bray-Curtis R = 0.071, *p* = 0.339; Unweighted Unifrac R = 0.0397, *p* = 0.475).
(TIF)

S11 Fig β-diversity by Bray-Curtis and Unweighted Unifrac of wild *T*. *infestans* infected and uninfected with *T*. *cruzi* and visualized with PCoA. Infected wild insects are shown in red and uninfected wild insects are shown in blue. Whilst the visual inspection of the two PCoAs do not indicate that *T*. *cruz*i infection associates with β-diversity among wild insects, the non-parametric permutation ANOSIM tests found evidence of clustering in β-diversity between infected and uninfected wild insects, with 1000 permutations (Bray-Curtis R = 0.250, *p* = 0.021; Unweighted Unifrac R = 0.215, p< = 0.012).
(TIF)
