# Supplementary material for: Hindgut microbiota in laboratory-reared and wild Triatoma infestans
Source: PLoS Negl Trop Dis. 2019 May 6;13(5):e0007383. doi: 10.1371/journal.pntd.0007383 (PMC6522061; doi:10.1371/journal.pntd.0007383)
Supplement: S1 Table — (DOCX) [file pntd.0007383.s001.docx]

**S1 Table. Baseline characteristics of *T. infestans* samples in this study.**

|  | **Lab-reared n (%)** | **Wild-caught n (%)** |
| --- | --- | --- |
| **n** | 10 | 59 |
|  |  |  |
| ***T. cruzi*-infected n (%)** | 4 (40.0) | 9 (15.3) |
|  |  |  |
| **Month and Year of Collection n (%)** |  |  |
| November 2011 | 0 | 2 (3.4) |
| Uninfected | - | 2 (3.4) |
| Infected | - | 0 |
| December 2011 | 0 | 12 (20.3) |
| Uninfected | - | 9 (15.3) |
| Infected | - | 3 (5.1) |
| January 2012 | 0 | 2 (3.4) |
| Uninfected | - | 2 (3.4) |
| Infected | - | 0 |
| February 2012 | 0 | 2 (3.4) |
| Uninfected | - | 2 (3.4) |
| Infected | - | 0 |
| March 2012 | 0 | 16 (27.1) |
| Uninfected | - | 16 (27.1) |
| Infected | - | 0 |
| April 2012 | 0 | 9 (15.3) |
| Uninfected | - | 9 (15.3) |
| Infected | - | 0 |
| May 2012 | 0 | 9 (15.3) |
| Uninfected | - | 9 (15.3) |
| Infected | - | 0 |
| August 2013 | 7 (70.0) | 0 |
| Uninfected | 3 (30.0) | - |
| Infected | 4 (40.0) | - |
| August 2015 | 3 (30.0) | 7 (11.9) |
| Uninfected | 3 (30.0) | 1 |
| Infected | 0 | 6 (10.2) |
| **District of Collection** |  |  |
| **Urban laboratory** | 10 (100.0) | 0 |
| **Urban and Periurban** |  |  |
| Alto Selva Alegre (A.S.A) | 0 | 32 (54.2) |
| Uninfected | - | 30 (50.8) |
| Infected | - | 2 (3.4) |
| Cayma | 0 | 1 (1.7) |
| Uninfected | - | 0 |
| Infected | - | 1 (1.7) |
| Hunter | 0 | 11 (18.6) |
| Uninfected | - | 11 (18.6) |
| Infected | - | 0 |
| Tiabaya | 0 | 1 (1.7) |
| Uninfected | - | 0 |
| Infected | - | 1 (1.7) |
| Yura | 0 | 8 (13.6) |
| Uninfected | - | (13.6) |
| Infected | - | 0 |
| **Rural districts** |  |  |
| Murco | 0 | 6 (10.2) |
| Uninfected | - | 1 (1.7) |
| Infected | - | 5 (8.5) |
|  |  |  |
| **Developmental Stage** |  |  |
| 3^rd^ instar | 0 | 6 (10.2) |
| Uninfected | - | 6 (10.2) |
| Infected | - | 0 |
| 4^th^ instar | 10 (100.0) | 7 (11.9) |
| Uninfected | - | 7 (11.9) |
| Infected | - | 0 |
| 5^th^ instar | 0 | 17 (28.8) |
| Uninfected | - | 11 (18.6) |
| Infected | - | 6 (10.2) |
| adult | 0 | 29 (49.2) |
| Uninfected | - | 23 (39.0) |
| Infected | - | 3 (5.1) |
