# Supplementary figures and images for: Hindgut microbiota in laboratory-reared and wild Triatoma infestans
Source: PLoS Negl Trop Dis. 2019 May 6;13(5):e0007383. doi: 10.1371/journal.pntd.0007383 (PMC6522061; doi:10.1371/journal.pntd.0007383)

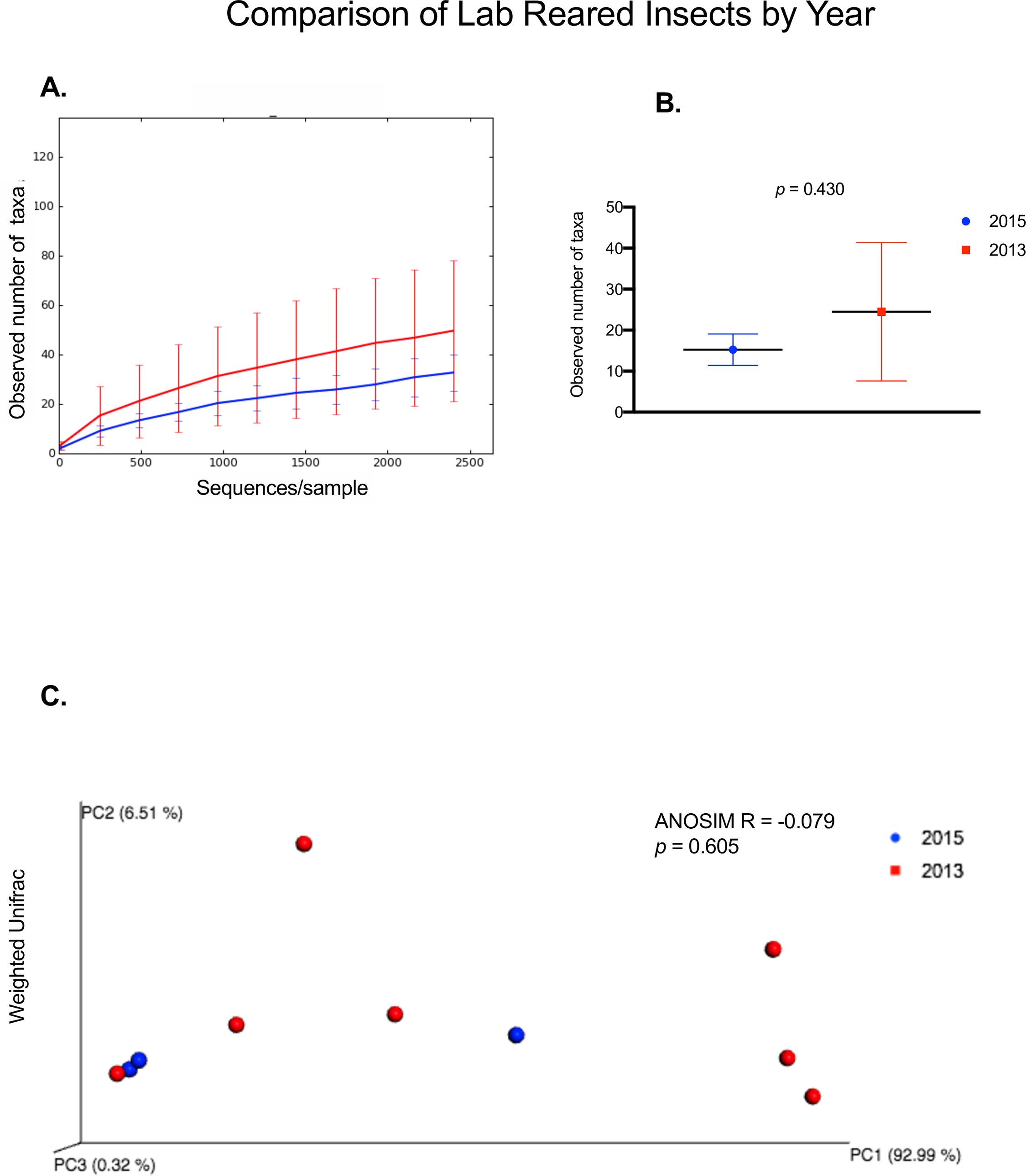

Supplement: S1 Fig — Panel A and B. The microbial composition of 2013 and 2015 insects did not differ significantly when comparing α-diversity with number of observed taxa as the metric (p = 0.430). Panel C. β-diversity (weighted Unifrac, ANOSIM R = -0.079, p = 0.605) did not differ significantly between 2013 and 2015 laboratory-reared insects. For subsequent analyses, 2013 and 2015 bugs were analyzed together. (TIF) [file pntd.0007383.s004.tif]

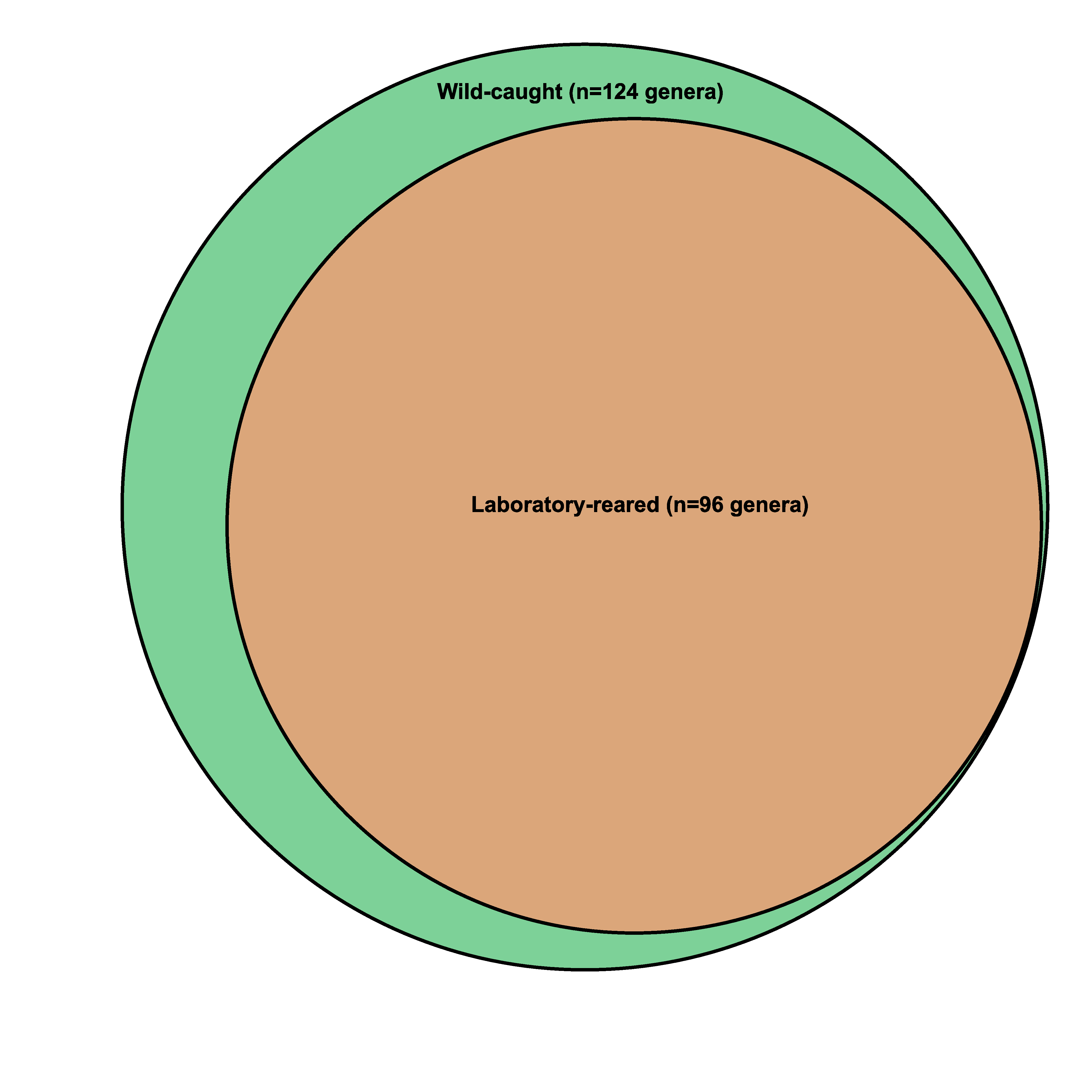

Supplement: S2 Fig — A total of 124 genera were identified in the complete dataset. All of the 124 genera were seen in the wild-caught T. infestans. Of these 124 genera, 96 of these were also seen in laboratory-reared T. infestans. (TIF) [file pntd.0007383.s005.tif]

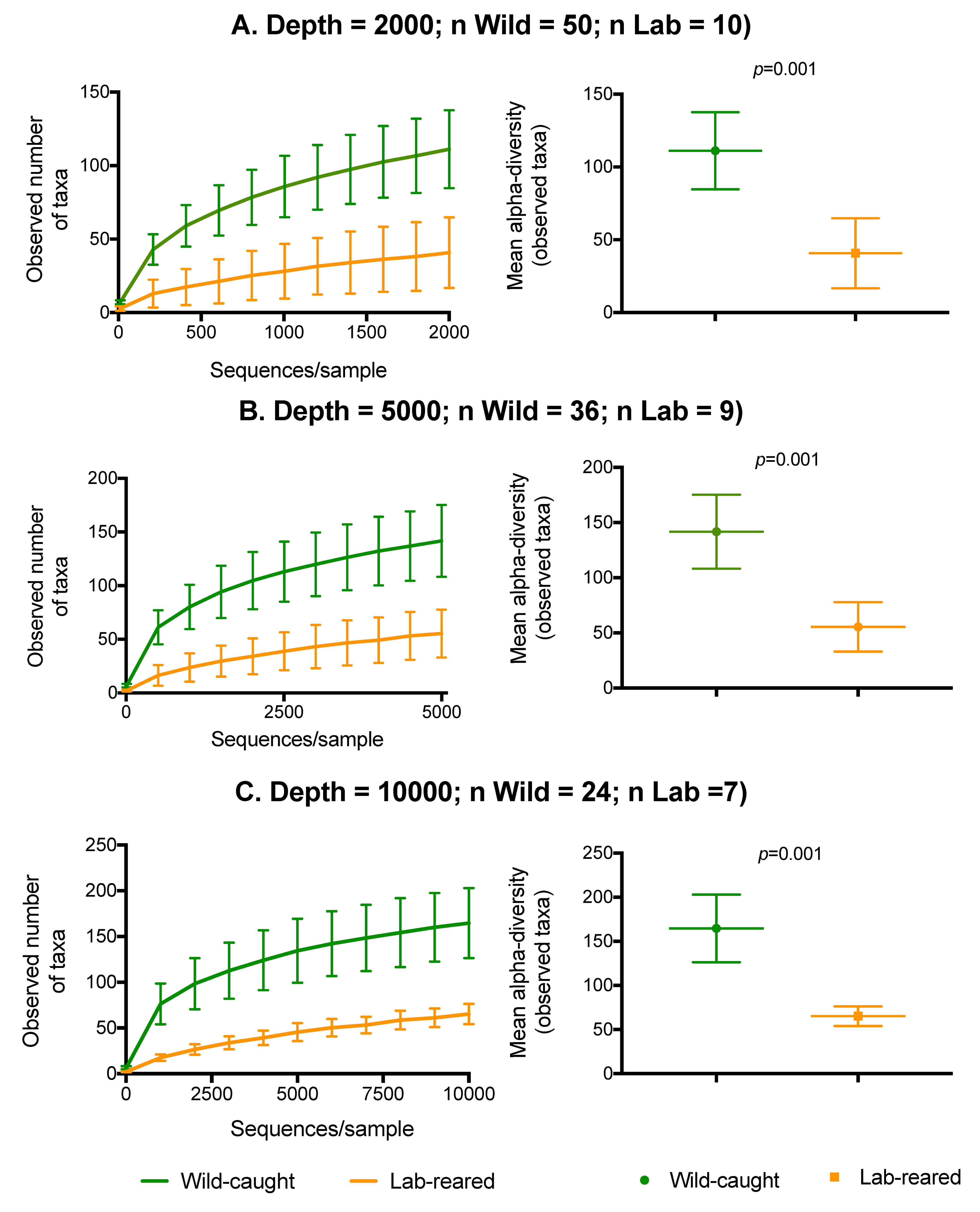

Supplement: S3 Fig — The number of observed taxa in lab insects was consistently lower than that in wild-caught insects (p<0.001), irrespective of sequence depth, indicating that a sequence depth of 2000 sequences for α-diversity analyses is adequate. (TIF) [file pntd.0007383.s006.tif]

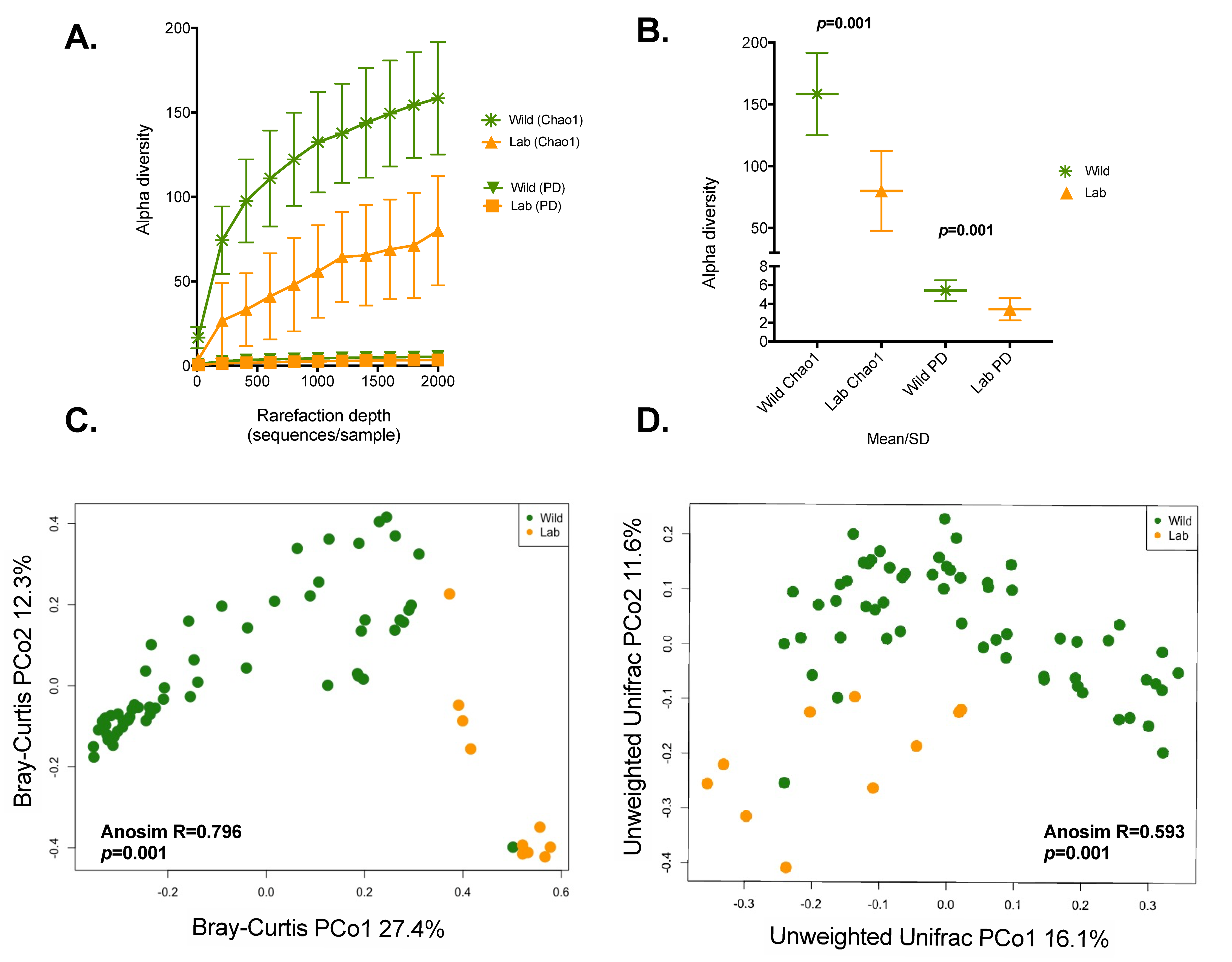

Supplement: S4 Fig — Wild insects are shown in green and lab insects are shown in orange. Panels A and B. α-diversity (Panel A) of laboratory-reared and wild-caught T. infestans was investigated at a depth of 2000 sequences/samples with Chao1 and Faith’s Phylogenetic Diversity (PD) as metrics. The α-diversity of wild and lab insects’ microbiota was then compared by averaging the iterations of rarefactions within sample group and then using non-parametric two-sample t-tests with Monte Carlo permutations to calculate the p-values (Panel B). Panels C and D. β-diversity of laboratory-reared and wild-caught T. infestans was computed by Bray-Curtis (Panel C) and Unweighted Unifrac (Panel D) and visualized by PCoA. Bray-Curtis, an abundance-based analysis, indicates separation between wild and lab microbial composition which may be due to differentially abundant taxa which exist between the two groups (refer to S5 Fig). Occurrence-based analysis Unweighted Unifrac also show separation between the wild and lab-reared insects because the microbial composition of lab bugs is a subset of that of wild bugs. These results of the Bray-Curtis and Unweighted Unifrac analyses are by non-parametric permutation ANOSIM tests which found significant β-diversity clustering between the lab and wild insects, with 1000 permutations (Bray-Curtis R = 0.796, p<0.001; Unweighted Unifrac R = 0.593, p<0.001). (TIF) [file pntd.0007383.s007.tif]

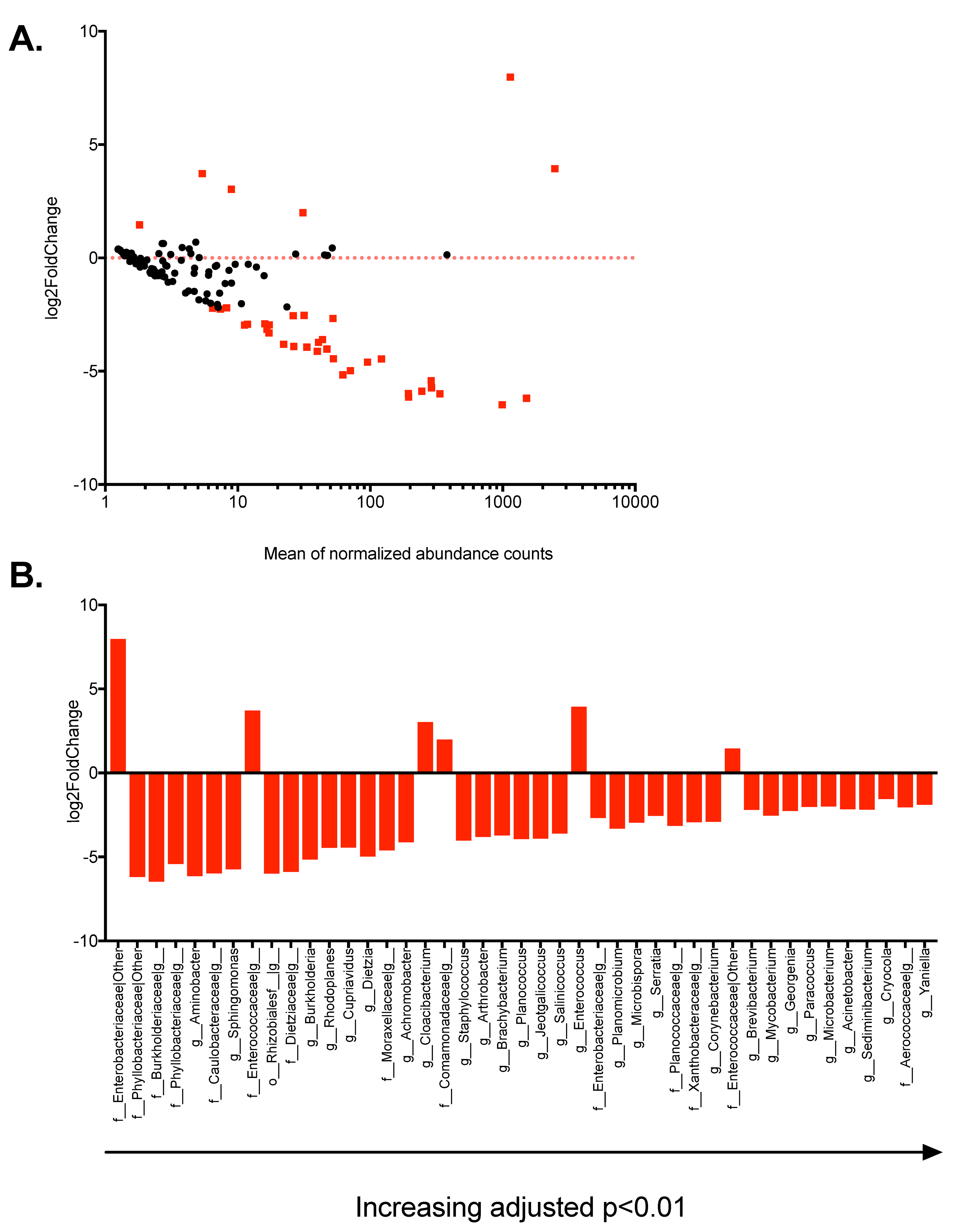

Supplement: S5 Fig — A. MA-plot (an application of Bland-Altman plot) showing the difference in relative abundance of taxa between lab and wild insects (log2 fold differences). Each point represents one of the 96 taxa common to both the lab and wild insects. Above the line (positive values of abundance differences) depict taxa which were overrepresented in laboratory insects compared to wild insects, and values below the line (negative differences in relative abundances) depict taxa which were underrepresented in lab insects compared to wild insects. Points are colored red if the adjusted p value is less than 0.01. A total of 43 of the 96 genus-level taxa common to both lab and wild insects were found to be differentially abundant (i.e. log2 fold change departing from 0) with p<0.01. B. The genus-level taxonomic classification of 43 of 96 taxa common to both lab and wild insects which were differentially abundant in the above analysis. The log2 fold difference in relative abundances in lab compared to wild insects is given on the y-axis. Taxa are ordered in increasing p-value from left to right (range = 2.392−45–0.009). Similarly to the above graph, points above the line (positive differences in relative abundances) depict taxa which were overrepresented in laboratory insects compared to wild insects, and values below the line (negative differences in relative abundances) depict taxa which were underrepresented in lab insects compared to wild insects. (TIF) [file pntd.0007383.s008.tif]

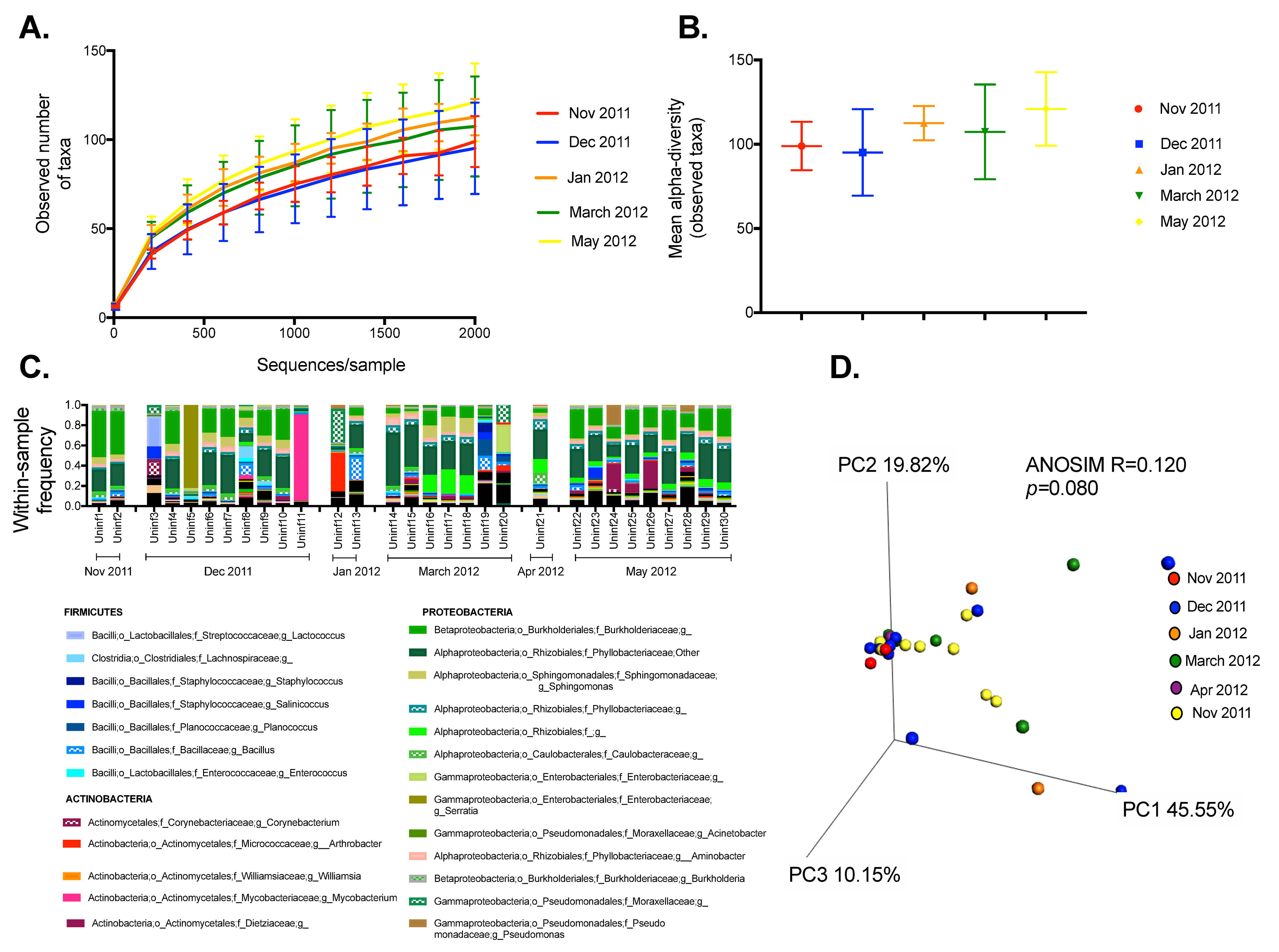

Supplement: S6 Fig — A total of 30 uninfected T. infestans were collected from A.S.A (Alto Selva Alegre) District across different months from November 2011 to May 2012. Panels A and B. The total number of observed taxa did not differ among insects collected during different months (p = 1.000 for all pairwise comparisons of observed taxa). There was only one insect captured during the month of April, and this sample was excluded from α-diversity analyses. Panels C and D. Heterogeneity in taxonomic composition was observed among insects collected during the same month, which can be gleaned from the bar plots of relative abundance (C) and in weighted Unifrac analysis and PCoA (D). Overall, month of collection was not a driver of diversity in the uninfected T. infestans collected from A.S.A. District. (TIF) [file pntd.0007383.s009.tif]

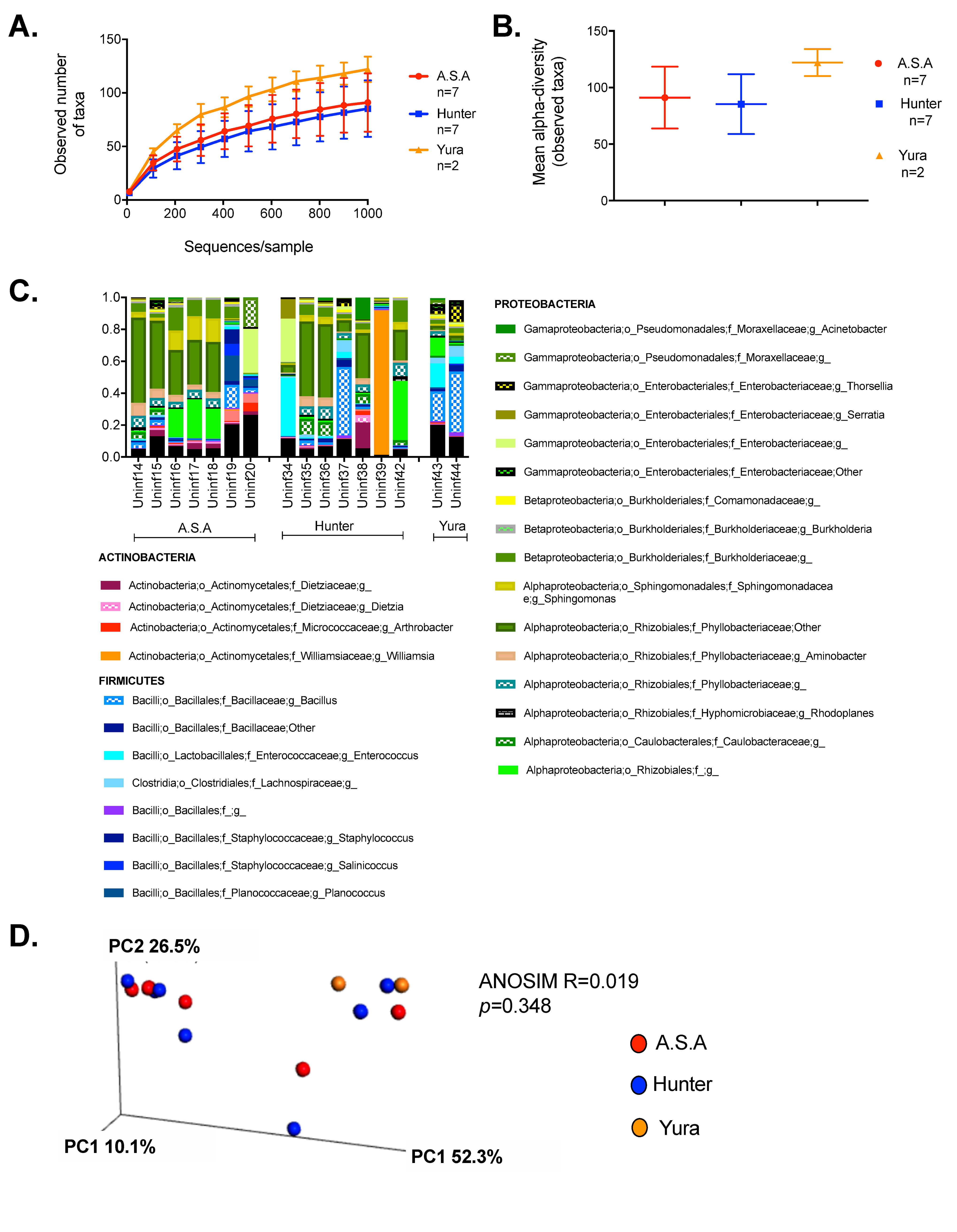

Supplement: S7 Fig — In the same month of March 2012, a total of 16 uninfected wild insects were collected from three districts (Alto Selva Alegre, A.S.A n = 7; Hunter n = 7; and Yura n = 2). Panels A and B. The α-diversity did not differ by district of capture when total number of observed OTUs was interrogated (range = 0.960–1.000 for the pairwise comparisons). Panels C and D. β-diversity analyses (weighted Unifrac and visualization by PCoA) also showed that district of capture was not a driver of the variation among insects and heterogeneity within the district was at times a stronger factor contributing in driving this variation, as was the case for sample Uninf20 and Uninf39. (TIF) [file pntd.0007383.s010.tif]

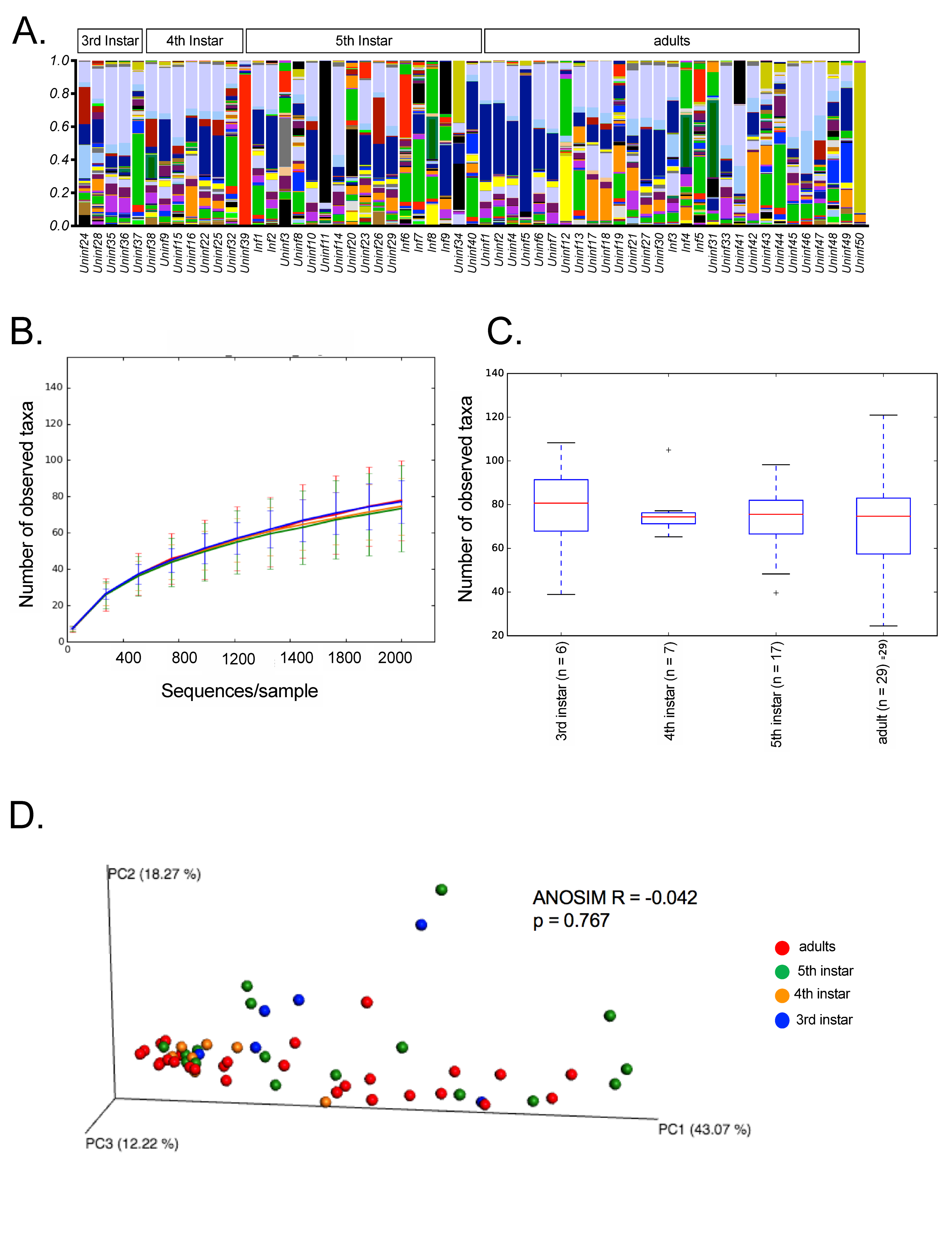

Supplement: S8 Fig — Panel A. Taxa plots of wild T. infestans sorted by developmental stage. Each vertical bar represents the microbial composition at the genus level (wherever unambiguous GreenGenes taxonomic classification at the genus level was possible), of each sample, and the within-sample frequency is denoted by the y-axis. Panels B and C. α-diversity by number of observed taxa of wild T. infestans third instars, fourth instars, fifth instars and adults. Panel B shows the rarefaction curves of the observed number of taxa of each insect group at a depth of 2,000 sequences and Panel C shows the comparisons of α-diversity between each insect group (by averaging the iterations of rarefactions within sample group and then using non-parametric two-sample t-tests with Monte Carlo permutations to calculate the p-values). Developmental stage did not associate with α-diversity measures (p = 1.000 among all comparisons). Panel D. β-diversity (weighted Unifrac) of wild T. infestans, by developmental stage. β-diversity was estimated with weighted Unifrac and formally tested for differences among insects of different developmental stages using the ANOSIM test. Variations patterns were visualized with PCoA. Developmental stage did not associate with variations in β-diversity of wild T. infestans (ANOSIM = -0.042, p = 0.767). (TIF) [file pntd.0007383.s011.tif]

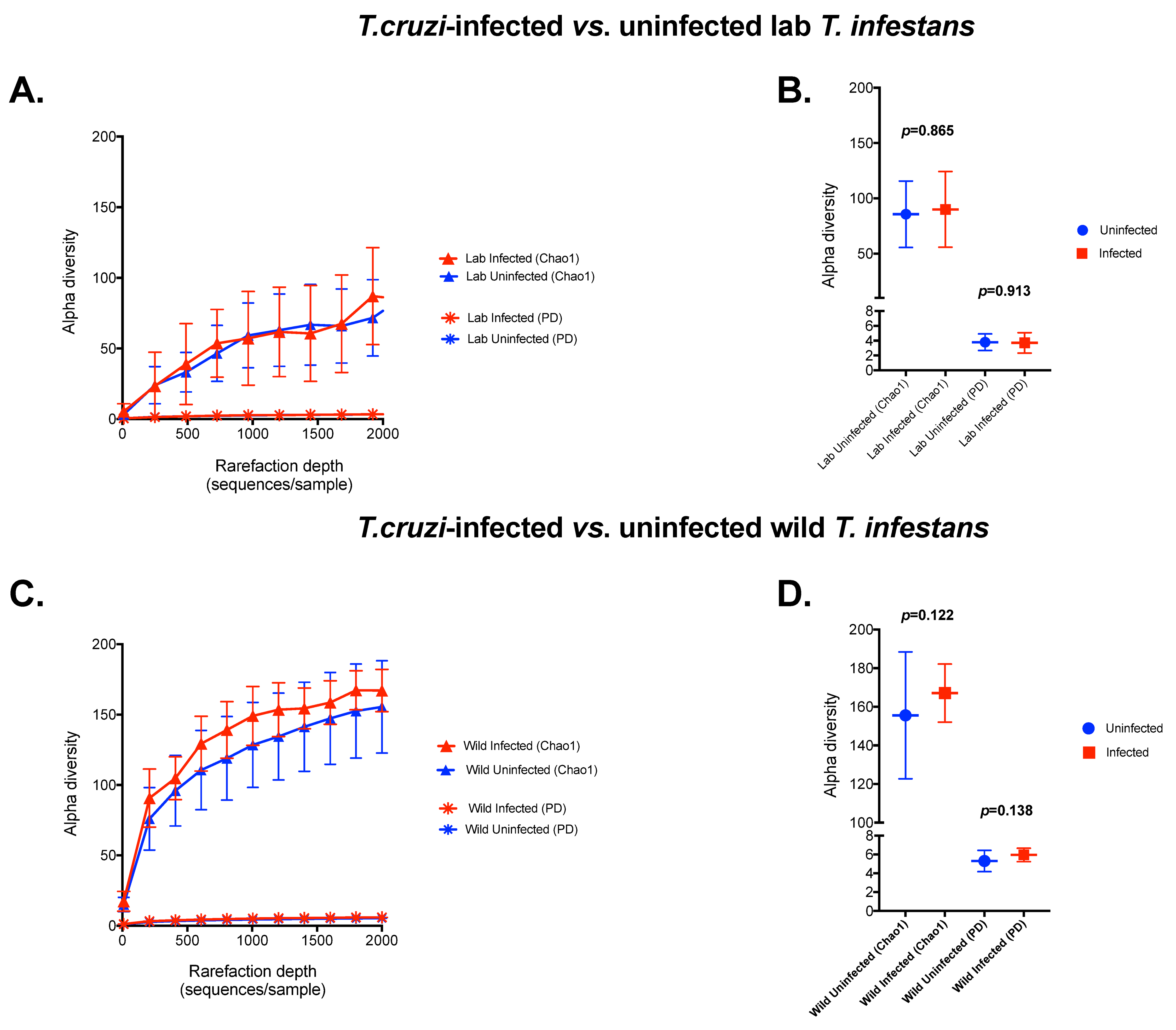

Supplement: S9 Fig — The presence of T. cruzi infection was confirmed by qPCR and microscopy in both lab and wild insects. Infected insects are shown in red and uninfected insects are shown in blue. Panels A and B. Lab-reared T. infestans. α-diversity was investigated at a depth of 2000 sequences/samples with Chao1 and Faith’s Phylogenetic Diversity (PD) as metrics (Panel A). The α-diversity of T. cruzi-infected and uninfected lab insects’ microbiota was then compared by averaging the iterations of rarefactions within sample group and then using non-parametric two-sample t-tests with Monte Carlo permutations to calculate the p-values (Panel B). α-diversity did not associate with T. cruzi infection in lab-reared insects (Chao1 p = 0.865, PD p = 0.913). Panels C and D. Wild-caught T. infestans. As for lab insects, α-diversity was investigated at a depth of 2000 sequences/samples (Panel C), then compared by averaging the iterations of rarefactions within sample group and non-parametric two-sample t-tests with Monte Carlo permutations to calculate the p-values (Panel D). In wild insects, α-diversity did not associate with T. cruzi infection (Chao1 p = 0.122, PD p = 0.138). (TIF) [file pntd.0007383.s012.tif]

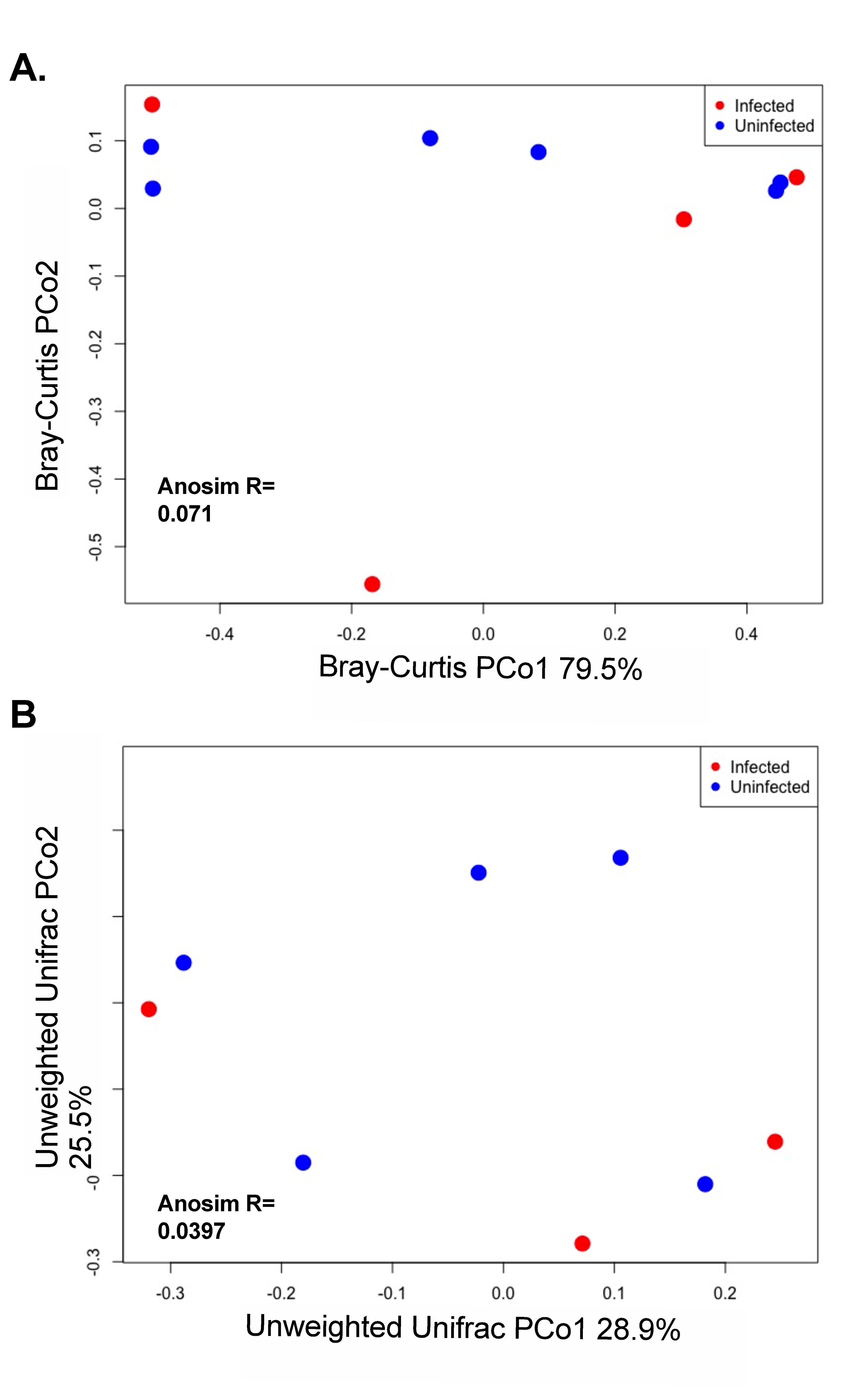

Supplement: S10 Fig — β-diversity was computed by Bray-Curtis (Panel A) and Unweighted Unifrac (Panel B) and visualized by PCoA. The results do not indicate that the microbiotas of infected and uninfected lab-reared insects are significantly different. This finding is supported by non-parametric permutation ANOSIM tests of the Bray-Curtis and the Unifrac β-diversity, which did not find significant β-diversity clustering between the infected and uninfected insects, with 1000 permutations (Bray-Curtis R = 0.071, p = 0.339; Unweighted Unifrac R = 0.0397, p = 0.475). (TIF) [file pntd.0007383.s013.tif]

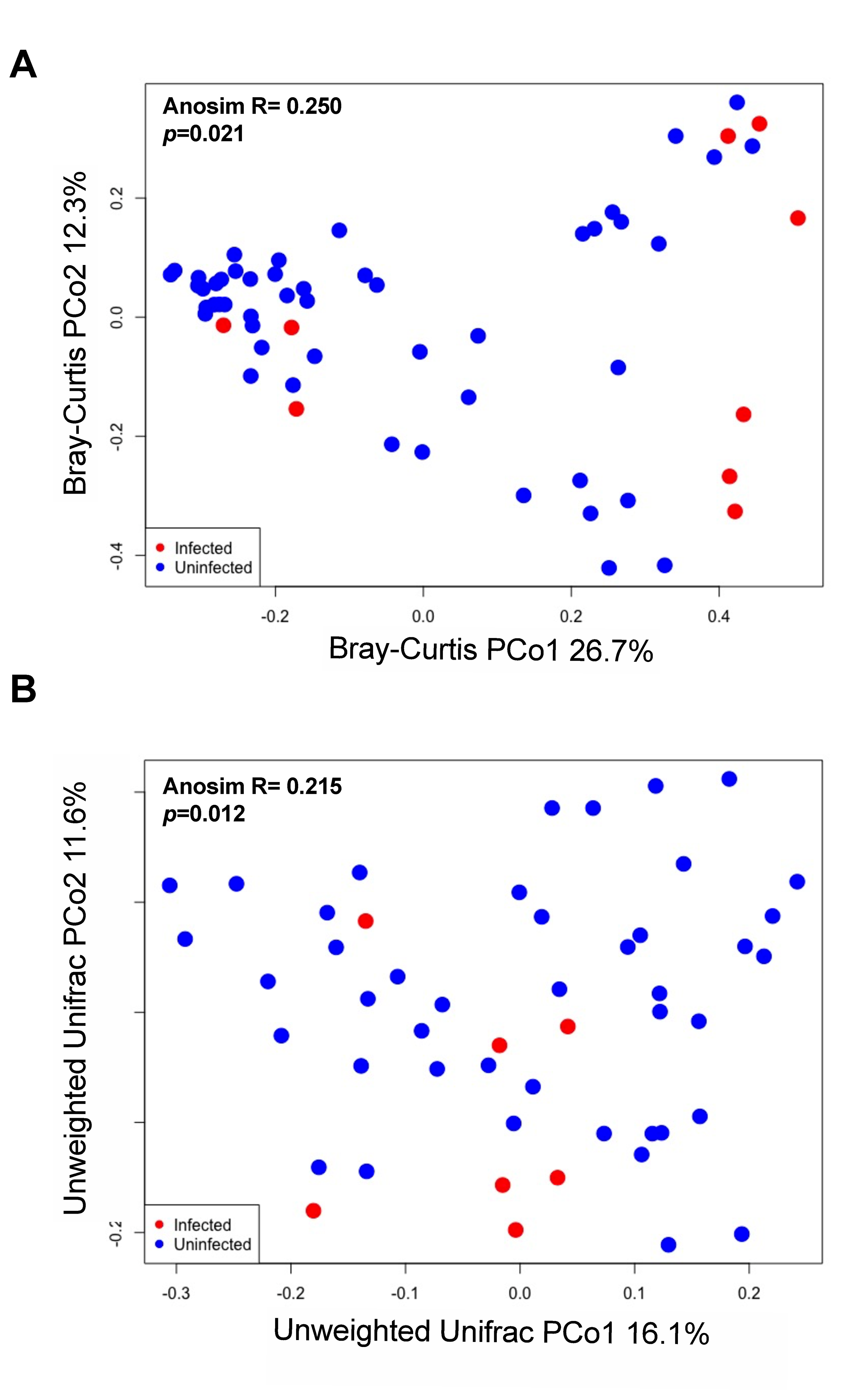

Supplement: S11 Fig — Infected wild insects are shown in red and uninfected wild insects are shown in blue. Whilst the visual inspection of the two PCoAs do not indicate that T. cruzi infection associates with β-diversity among wild insects, the non-parametric permutation ANOSIM tests found evidence of clustering in β-diversity between infected and uninfected wild insects, with 1000 permutations (Bray-Curtis R = 0.250, p = 0.021; Unweighted Unifrac R = 0.215, p< = 0.012). (TIF) [file pntd.0007383.s014.tif]
